# Bee species checklist of the San Francisco Peaks, Arizona

**DOI:** 10.3897/BDJ.8.e49285

**Published:** 2020-04-02

**Authors:** Lindsie M McCabe, Paige R Chesshire, David R Smith, Atticus Wolf, Jason Gibbs, Terry L Griswold, Karen W Wright, Neil S Cobb

**Affiliations:** 1 Department of Biological Sciences, Northern Arizona University, Flagstaff, United States of America Department of Biological Sciences, Northern Arizona University Flagstaff United States of America; 2 U.S. Fish and Wildlife Service, Southwest Forest Science Complex, Flagstaff, United States of America U.S. Fish and Wildlife Service, Southwest Forest Science Complex Flagstaff United States of America; 3 Department of Entomology, University of Manitoba, Winnipeg, Canada Department of Entomology, University of Manitoba Winnipeg Canada; 4 USDA-ARS, Pollinating Insects Research Unit, Logan, United States of America USDA-ARS, Pollinating Insects Research Unit Logan United States of America; 5 Department of Entomology, Texas A&M, College Station, United States of America Department of Entomology, Texas A&M College Station United States of America

**Keywords:** Northern Arizona, Southwestern, United States, Bee Diversity, Faunistics, Elevation Gradient, Anthophila

## Abstract

**Background:**

Here we present a checklist of the bee species found on the C. Hart Merriam elevation gradient along the San Francisco Peaks in northern Arizona. Elevational gradients can serve as natural proxies for climate change, replacing time with space as they span multiple vegetation zones over a short geographic distance. Describing the distribution of bee species along this elevation gradient will help predict how bee communities might respond to changing climate. To address this, we initiated an inventory associated with ecological studies on pollinators that documented bees on the San Francisco Peaks. Sample sites spanned six life zones (vegetation zones) on the San Francisco Peaks from 2009 to 2019. We also include occurrence data from other studies, gathered by querying the Symbiota Collection of Arthropods Network (SCAN) portal covering the San Francisco Peaks region (hereafter referred to as “the Peaks”).

**New information:**

Our checklist reports 359 bee species and morphospecies spanning five families and 46 genera that have been collected in the Peaks region. Prior to our concerted sampling effort there were records for 155 bee species, yet there has not been a complete list of bee species inhabiting the Peaks published to date. Over a 10-year period, we documented an additional 204 bee species inhabiting the Peaks. Our study documents range expansions to northern Arizona for 15 species. The majority of these are range expansions from either southern Arizona, southern Utah, or the Rocky Mountain region of Colorado. Nine species are new records for Arizona, four of which are the southernmost record for that species. An additional 15 species are likely undescribed.

## Introduction

The North American Southwest has one of the highest biodiversity of bee species worldwide (Michener 1979), with Arizona in particular harboring over 1,500 bees spanning six different families (SCAN 2019). This is largely due to the wide habitat diversity within such a short geographic distance, ranging from desert ecosystems to high-elevation mountain environments. The elevational gradients that characterize “Sky Islands” (i.e. isolated mountain tops) are key biodiversity hotspots in the Southwest (Bowers and McLaughlin 1996). Due to the isolated nature of sky islands, the biota of these unique geographic areas is acutely susceptible to climate change.

In northern Arizona, the San Francisco Peaks region (hereafter referred to as “the Peaks”) is one of the northern most sky islands and is characterized by the C. Hart Merriam elevational gradient, ranging from 785 to 3,850 meters (Merriam 1890). This range of life zones includes habitats of low-elevation desert ecosystems, high-elevation forest types, and environments above-tree-line. This variation is created by a steep gradient of temperature and precipitation. Elevational gradients are attractive for studies of global climate change by exchanging time for space and are useful in a comparative sense for understanding latitudinal patterns (Blois et al. 2013). Despite inherent constraints in using elevational gradients as proxies for latitudinal gradients or climate change, they remain a focus of research interests in understanding ecological patterns and processes and are a high priority for conservation.

There have been multiple checklists published within the last year summarizing the bee species found in various regions of North America, including areas in the southern and western US (Messinger and Griswold 2002, Carril et al. 2018, Parys et al. 2018, Stephenson et al. 2018, Delphia et al. 2019, Meiners et al. 2019). However, there are no published checklists for northern Arizona. This is the first checklist published for the Peaks and is of special interest because it includes distributions of native bees along an elevational gradient with diverse habitats. More localized studies are necessary in order to obtain baseline knowledge on distributions and species richness of North American bee communities (Jamieson et al. 2019). If species trends and distributions are known regionally, we can better predict how native bee ranges and population statuses may be affected with changing climate.

## Materials and methods

### Study site and collection methods

**Figure 1.**
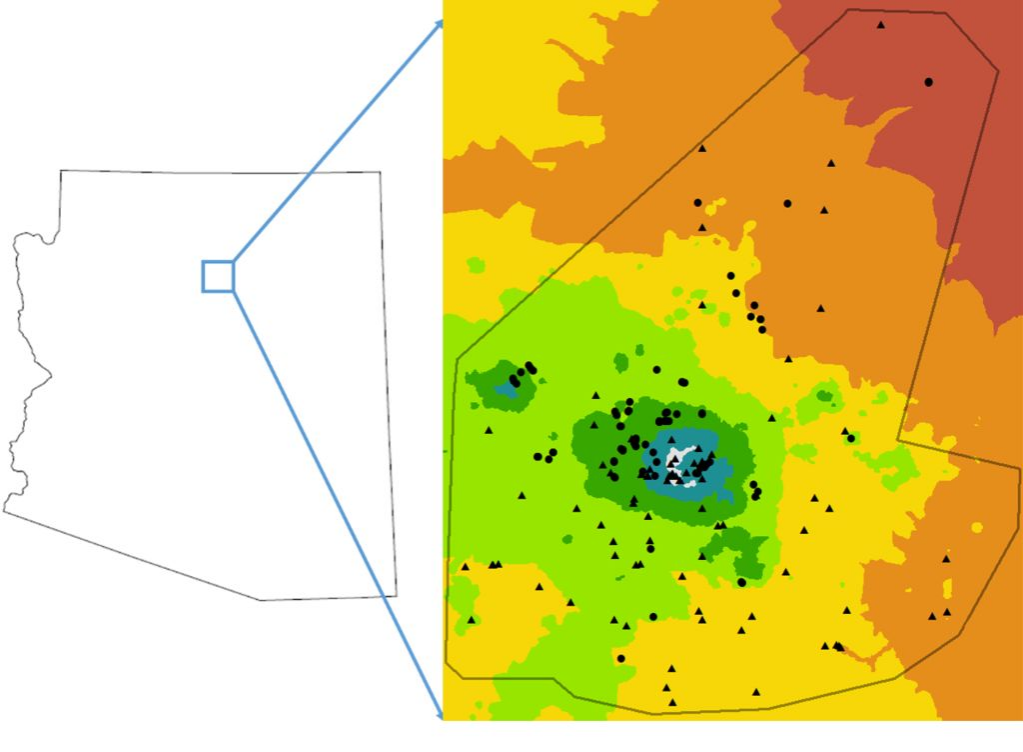
Map of collection instances on the San Francisco Peaks with life zones coded by color (dark red = desert shrub, orange = desert grassland, yellow = pinyon-juniper, green = ponderosa pine, dark green = mixed conifer, blue = spruce-fir and white = alpine). Black dots indicate our 58 survey plots from 2009–2019. Black triangles represent any unique collection instance gathered through SCAN, GBIF & iDigBio.

Research was conducted on the San Francisco Peaks in northern Arizona (Fig. 1). A total of fifty-eight sites were established across six distinct life zones (Table 1). The Peaks is the northern most mountain habitat in Arizona, consisting of a range of habitats from desert to alpine environments. Our study area consisted of six vegetation zones classified by the dominant vegetation type: desert shrub, desert grassland, pinyon-juniper forest, ponderosa pine forest, mixed conifer forest (dominated by aspens), and spruce-fir forest. We conducted three complementary studies 1) cup sampling from 2009–2012, 2) cup sampling from 2013–2016 and 3) flower sampling from 2016–2018. We also did qualitative (non-standardized) sampling along the Peaks in 2019. We created a reference collection of all bee species collected during the study.

***Cup Sampling: 2009–2012** (Sites: DS1, DG1, PJ8, PP5, MC2)*: Pollinators were sampled from 2009-2012 at five life zones ranging from desert shrub to mixed conifer, with one site established at each life zone. At each site we placed one pollinator cup array, which consisted of 30 pollinator cups (i.e. elevated pan traps). Each cup was filled with 50/50 water/propylene glycol about 2/3 of the way full. The pollinator cups were 12 oz. plastic stadium cups (10 white, 10 fluorescent yellow and 10 fluorescent blue). White, yellow and blue colors accounted for all of the major flora colors in this area (Campbell and Hanula 2007). The outside diameter of the cup opening was 8 cm, and the cups were 10.7 cm deep (McCabe et al. 2019b, Smith et al. 2014). Cups were suspended 30 cm above the ground in specially built holders made of polyvinyl chloride (PVC) pipes (Smith et al. 2014) to approximate the height of most flowering plants (Cane et al. 2000). Traps were placed in three rows of 10 (where each row was a single color), 10-m apart. Each cup within the row was placed 3-m apart. Traps were set once per month for 7 to 8 days. Cups did not become filled to the top with specimens throughout this time frame, so bees were consistently collected for the full 7 to 8 days. The two lower elevation sites, desert shrub and desert grassland, were sampled from April through October as freezing temperatures abated earlier at these sites than at higher elevations. Traps were set from May through October at the higher elevation sites (pinyon-juniper, ponderosa pine, and mixed conifer).

***Cup Sampling: 2013–2014** (Sites: PP1A-PP3A, PP1F-PP3F, MC1-MC3, MC1F-MC3F, SF1A-SF3A, SF1F-SF3F)*: Bees were sampled using pollinator cups at three life zones on the Peaks: ponderosa pine, mixed conifer and spruce-fir. We sampled at three unique sites at each life zone and set up pollinator arrays in two distinct locations per site: one array was placed in a meadow habitat and one was placed in a forest habitat. An array consisted of nine pollinator cups (three rows, each row with three cups of the same color). Details on our method of pollinator cup trapping is described above. Each year pollinator cups were set up during two seasons: dry pre-monsoon (June) and monsoon (August). During the monsoon season of 2013, 50% of the pre-monsoon cups were lost to animal damage at our Peaks sites at the spruce-fir elevation.

***Cup Sampling: 2015*** (*PP1-PP3, PP1A-PP3A, PP1F-PP3F, MC1-MC3, MC1F-MC3F, SF1-SF3, SF1A-SF3A, SF1F-SF3F)*: Cup sampling methods were identical to those used in 2013-2014, however we added an additional three sites at both ponderosa pine and spruce-fir (PP1-PP3, SF1-SF3). In addition, we established pollinator cup arrays on Kendrick Mountain, a neighboring mountain within the Peaks region, where we sampled at three life zones: ponderosa pine, mixed conifer, and spruce-fir, with three sites at each life zone *(KEN1A-KEN3A, KEN1B-KEN3B, KEN1C-KEN3C)*. Cup sampling methods and array design were identical to that used for the cup sampling on the Peaks. Each year, for both mountains, pollinator cups were set up during two seasons: dry pre-monsoon (June) and monsoon (August).

***Cup Sampling: 2016** (PJ1-PJ8, PP1-PP8, MC1-MC8, SF1-SF8)*: Cup sampling methods were identical to those used in 2013-2015, however there were differences in the sampling sites. Some sites were reused from previous years (PJ8, PP1-PP3, MC1-MC3, SF1-SF3). An additional five sites were established at each of the three higher life zones (PP4-PP8, MC4-MC8, SF4-SF8), and seven new sites were established at the pinyon juniper ife zone (PJ1-PJ7). This led to a total of 32 sites, with eight sites per life zone.

***Flower Sampling: 2016–2018** (Sites: PJ1-PJ8, PP1-PP8, MC1-MC8, SF1-SF8)*: In 2016, transect plots were established at four life zones: pinyon-juniper, ponderosa pine, mixed conifer, and spruce-fir. Eight sites were established at each life zone that were at least 1 km apart, with each site containing three 60-meter × 1-meter transects. Five sites were re-used from previous sampling years (PJ8, PP5, MC1, MC2, and MC3). Using modified hand vacuums (Lance et al. 2017), insects were collected directly from flowers for 15 minutes at each transect. Sampling periods occurred every two weeks from June to August. In 2017 and 2018, the transects established at each site were expanded to 60-meter × 2-meter plots, and insects were collected from flowers for 30 minutes at each transect.

***Flower Sampling: 2019***: Qualitative sampling was done in 2019. Bees were collected off of flowering plants using sweep nets near the base of Mount Eldon (considered ponderosa pine life zone) as well as near Snowbowl Ski Resort (considered mixed conifer and spruce-fir life zones). A few additional specimens were collected at sites used in previous years (PP5, MC1, MC5, SF3, SF6). Latitude and longitude decimal points for all 2019 sampling locations are provided (Suppl. material 1).

A total of 6,324 cups and 128 flower sampling hours were used in this data set.

### Species identification

All bees collected in samples were curated and initially identified in the Northern Arizona University (NAU) pollinator ecology lab. Bees were identified using DiscoverLife.org and published identification guides. Classification for species of *Andrena* and *Melissodes* followed LaBerge (1969), LaBerge (1986), LaBerge (1967)with modifications from Karen Wright's work, and all other species followed the classification of Michener (2000). Genus-level identifications were done using the Bee Genera IDnature guide from DiscoverLife.org (Ascher and Pickering 2011) and The Bee Genera of North and Central America (Michener et al. 1994). Species-level identification was done using published literature (Sandhouse 1924, Michener 1938, Michener 1939, Michener 1947, Hurd and Linsley 1951, Timberlake 1952, Stephen 1954, Timberlake 1954, Hurd and Michener 1955, Snelling 1966, LaBerge 1967, LaBerge 1969, Roberts 1972, Daly 1973, McGinley 1986, LaBerge 1986, Michener et al. 1994, Michener 2000, Sipes 2002, Rightmyer 2008, Gibbs 2010, Rightmyer et al. 2010, Sheffield et al. 2011, Koch 2012, De Silva and Packer 2012, Gonzalez and Griswold 2013, Robertson et al. 2014, Williams et al. 2014) and confirmed by Terry Griswold, Harold Ikerd (Andrenidae), Jason Gibbs (*Lasioglossum*) and Karen Wright (*Melissodes)*. Vouchers were deposited in the Colorado Plateau Museum of Arthropod Biodiversity, ARS Pollinating Insect Research Unit, Wallis Roughley Museum of Entomology, and Texas A&M University Insect Collection.

For those genera or subgenera where taxonomic information was lacking, we classified bees with similar morphological distinctions into morphospecies. Each morphospecies is classified by the genus (and subgenus if determined) followed by a unique three-digit number. Male and female specimens of the same morphospecies were combined. Species that were morphologically different were treated as unique morphospecies. All morphospecies listed are all potentially undescribed taxa.

We established a reference collection of bee species that is currently stored in the Colorado Plateau Museum of Arthropod Biodiversity at NAU. All specimens were digitally cataloged in the Symbiota Collections of Arthropods Network (SCAN) online data portal. Identification of the 65 species that were not collected by the NAU lab and confirmed by NAU, the Logan Bee Lab, Jason Gibbs or Karen Wright need further consideration, especially in instances/localities where they have not been collected for 20+ years. These 65 taxa are noted with the year that they were last collected on the Peaks. Further, one-third of these taxa (20 species) were not assigned to a life zone due to a lack of precision in the latitude and longitude coordinates. These 20 species were removed from further analysis (Suppl. material 2). There were additional 68 species that had records with imprecise latitude and longitude removed, however we could still assign life zone designations to these 68 species because there were other sampling instances where the localities were accurate (Suppl. material 2).

### Range

To determine species ranges, we used occurrence records from four main databases: SCAN, iDigBio (Integrated Digitized Biocollections), GBIF (Global Biodiversity Information Facility), and DiscoverLife. Species were deemed a new record for Arizona if there were not any previous records documented within the Arizona state boundaries on any of the four data portals mentioned above. We examined published literature to verify that these species were not previously recorded within the Arizona state boundary (Sandhouse 1924, Michener 1939, Sandhouse 1941, Timberlake 1969, LaBerge 1973, Roberts 1973, Krombein et al. 1979). Species were deemed a new record in northern Arizona if there were no records north of the Phoenix metropolitan area. We provide a KML map that defines our study area on the Peaks that is outlined in black (Fig. 1). We also provide a Darwin Core Archive (DwC) file of all records from our study area (Suppl. material 3).

Species were assigned "notes" if 1) they had not been recorded in our study range prior to our 10-year NAU study or 2) they were not collected in our 10-year NAU study but were collected in previous years from other sampling events (followed by the year that the species was last collected). Records obtained through SCAN, GBIF and iDigBio databases provided this information.

## Checklists

### Andrenidae (n = 72)

#### Andrena (Andrena) coconina

LaBerge, 1980

EE553594-CFF1-5C83-B19B-73804BDE5E40

##### Notes

Last collected on the Peaks in 1952

#### Andrena (Andrena) frigida

Smith, 1853

10FF2589-57E7-565C-958C-49FAF484AADF

##### Notes

Last collected on the Peaks in 1986

#### Andrena (Belandrena) 001

FCEDFE2B-0D94-5206-8C18-20B51588540F

#### Andrena (Callandrena sensu lato) helianthi

(Robertson, 1891)

B4BB85C9-7692-5FB4-B8B0-0226E2C544FF

#### Andrena (Callandrena sensu lato) pecosana

Cockerell, 1913

056650F2-725E-5D9B-A65D-A50C61DC4E66

#### Andrena (Callandrena sensu lato) sonorensis

LaBerge, 1967

1E322496-9272-5707-A468-CEADE384DD51

##### Notes

Last collected on the Peaks in 1976

#### Andrena (Callandrena) auripes

LaBerge, 1967

D57674BA-0323-597A-B835-F09BE76A2FBF

#### Andrena (Callandrena) micheneriana

LaBerge, 1978

99932799-3006-5D38-8B1E-981DE30DFE90

#### Andrena (Callandrena) perpunctata

LaBerge, 1967

3994EE18-CC22-5AC2-8FA6-C6EC2AD6ADE5

#### Andrena (Callandrena) simulata

Smith, 1879

4330DD37-635D-5EC9-857A-283B5478CAD0

#### Andrena (Callandrena) tegularis

LaBerge, 1967

3C77101A-900E-5B83-8AF1-B59BB96CBC69

#### Andrena (Cnemidandrena) apacheorum

Cockerell, 1897

B5BBC7A3-8950-5173-9ACB-5ECC0CC367D4

#### Andrena (Cnemidandrena) costillensis

Cockerell, 1914

E3FEF0B8-6F65-5D56-B34D-E394731B8B9D

#### Andrena (Cnemidandrena) nubecula

Smith, 1853

68E728AC-8DD3-577D-BA1F-D71661CC6E7B

#### Andrena (Diandrena) 001

Fabricius, 1775

67C51B3F-834B-54CC-BCB5-13F84759CFD7

#### Andrena (Euandrena) algida

Smith, 1853

568DF721-899F-50FE-8F0C-2274C5F2DC6B

#### Andrena (Holandrena) cressonii

(Robertson, 1891)

DE667109-A24C-5A68-B100-411B8960BF9B

##### Distribution

Our record is the first documentation of this species in northern Arizona. Species occurs in neighboring areas.

#### Andrena (Holandrena) moquiorum

Viereck & Cockerell, 1914

2EA72269-F9CE-50D6-9DE4-6430570D3D22

##### Notes

Last collected on the Peaks in 1902

#### Andrena (Melandrena) commoda

Smith, 1879

700CE654-CF4D-5C48-B5F5-605F5E77D1BF

#### Andrena (Melandrena) crinita

Bouseman & LaBerge, 1979

0C07EB11-8C64-5C6A-A3DD-72CFCCABD514

#### Andrena (Melandrena) platyrhina

Cockerell, 1930

0797F720-36D2-55E1-B241-6BDCAC7C1D9D

#### Andrena (Plastandrena) argemonis

Cockerell, 1896

8F3E4AAE-633B-599F-9FD7-9CE4927DBD74

#### Andrena (Plastandrena) crataegi

Robertson, 1893

FF2B235A-AEAC-5B35-926A-40A91CD1B1EE

#### Andrena (Plastandrena) prunorum

Cockerell, 1896

ED0E9277-5D85-56BB-90F2-50ECD178A79D

#### Andrena (Simandrena) angustitarsata

(Viereck, 1904)

DCBB3B1E-36ED-5D22-B457-5A9C24BCDA36

#### Andrena (Thysandrena) medionitens

Cockerell, 1902

841FFBB1-E793-5E7C-BA22-A88C5B63770B

#### Andrena (Thysandrena) w-scripta

Viereck 1904

6D40E315-9042-5FBF-9FAF-0EDF2071BC24

#### Andrena (Trachandrena) amphibola

(Viereck, 1904)

9DAB65D5-0AE3-521B-A5C4-0D4C2E34F489

##### Distribution

Our records are the first documentation of this species in Arizona and the southernmost extension of its range. Species occurs in neighboring areas.

#### Andrena (Trachandrena) cyanophila

Cockerell, 1906

9E133523-1B84-57BF-B7F4-C90F9BE43508

#### Andrena (Trachandrena) mariae

(Robertson, 1891)

27D16DB7-9C8C-5A6C-9330-F8D09371DD8F

##### Distribution

Our record is the first documentation of this species in Arizona. Species occurs in neighboring areas.

#### Andrena (Trachandrena) miranda

Smith, 1879

4478B4E8-75B2-5204-863D-03007914CB8F

#### Andrena (Trachandrena) striatifrons

(Cockerell, 1897)

9D5EB134-49BA-5959-B60B-47F61EBAB150

#### Andrena (Trachandrena) 001

Robertson, 1902

7A325DBC-2149-53AC-B8ED-35F8F3EBD1AC

#### Andrena 001

Fabricius, 1775

EFA2D9EE-87F0-5E8E-B207-EC8EF0C3ACCB

#### Andrena 003

Fabricius, 1775

EF3FCD8B-548D-574D-843C-EC20245A629F

#### Andrena 004

Fabricius, 1775

BC10DF13-E9F1-5A81-98B2-3EE659988B9D

#### Andrena 005

Fabricius, 1775

8ABE8CA3-E2C0-5698-BDF7-2F93EDBF16EA

#### Andrena 006

Fabricius, 1775

E58F4BC1-E6A5-525C-A649-49B4B0525389

#### Calliopsis (Calliopsima) chlorops

Cockerell, 1899

6F5938D7-FE8E-5166-9BB4-85C1BC4AB355

#### Calliopsis (Calliopsima) rozeni

Shinn, 1965

DB5A2656-696A-5BFB-A0EF-59C68319E760

##### Notes

Last collected on the Peaks in 1951

#### Calliopsis (Calliopsis) teucrii

Cockerell, 1899

7DCA38EA-0BDD-59BA-AC76-4318DEFBA20D

##### Notes

Last collected on the Peaks in 1961

#### Calliopsis (Hypomacrotera) callops

(Cockerell and Porter, 1899)

52F4D3BE-C47B-5743-9DEF-979B5D2AC363

#### Calliopsis (Nomadopsis) puellae

(Cockerell, 1933)

72A91B0C-7F35-58D1-8AC7-196ED20C5DD5

#### Calliopsis (Nomadopsis) timberlakei

(Rozen, 1958)

77C27628-DB02-5C8D-A13B-F815B99BD568

#### Calliopsis (Nomadopsis) zebrata

(Cresson, 1878)

FF62439F-9B05-594B-8625-8628AC9C3227

#### Calliopsis 001

Smith, 1899

2E8A5D47-E8A7-5615-9000-8C72A195CE6B

#### Macrotera (Macroteropsis) latior

(Cockerell, 1896)

4B53C0FE-5DDF-5AF9-BB57-C2E8159FFBFB

#### Perdita (Epimacrotera) giliae

Timberlake, 1954

D3BA903F-89E5-585B-9E76-F380D5DA309C

##### Notes

Last collected on the Peaks in 1964

#### Perdita (Perdita) gutierreziae

Cockerell, 1896

7B31D55E-3C61-57B0-B0CF-9075E8DA1E88

##### Notes

Last collected on the Peaks in 1965

#### Perdita (Perdita) sphaeralceae

Cockerell, 1896

3BAE7D80-66A4-5A50-8A4F-6A7D1E602EA7

##### Notes

Last collected on the Peaks in 1967

#### Perdita (Perdita) zebrata

Cresson, 1878

05A7D981-951F-5CDE-836D-80F7E0A99041

##### Notes

Last collected on the Peaks in 1952

#### Perdita 001

Smith, 1853

8B97B4A9-C73F-52B2-8E9D-42F39C2BB14C

#### Perdita 002

Smith, 1853

FAA139D9-5498-5423-97DE-EBF99F502AAB

#### Perdita 003

Smith, 1853

C995971D-99E1-5005-9D55-D74BF7E532CA

#### Perdita 004

Smith, 1853

41551D8F-A56B-5278-8E74-F31832755B7E

#### Perdita 005

Smith, 1853

233873F0-F640-5EE1-9156-E2A5888E83F0

#### Perdita 006

Smith, 1853

A78FA619-8E98-5C8C-8B6C-05EE14BFDAC7

#### Perdita 007

Smith, 1853

21EFA0A9-DF26-507B-B6CD-30F0F277C08B

#### Perdita 008

Smith, 1853

16D25AEA-6330-5F24-A9C2-2C02E5402005

#### Perdita 009

Smith, 1853

52F34359-2036-5282-8C20-FEEB9827493F

#### Perdita 010

Smith, 1853

02B1A8EA-F937-5287-A410-0E4E341B7A4D

#### Protandrena (Heterosarus) neomexicanus

Cockerell, 1906

DDF4C8B3-0AED-535B-8B5A-520194FC088D

##### Notes

Last collected on the Peaks in 1958

#### Protandrena (Heterosarus) 001

Robertson, 1904

CC35BF5D-643A-579E-BE92-6AB414294EAA

#### Protandrena (Heterosarus) 002

Robertson, 1904

24FC68C2-808C-5A65-BB42-62C54D43197A

#### Protandrena (Heterosarus) 003

Robertson, 1904

D8DD6A0E-56A8-51C4-8054-8FEA79E9D944

#### Protandrena (Heterosarus) 004

Robertson, 1904

A6CEBAB5-0724-531B-A230-DBE70ECE6668

#### Protandrena (Heterosarus) 005

Robertson, 1904

520675AE-04BA-5260-B208-911B76741DEE

#### Protandrena (Pterosarus) albitarsis

(Cresson, 1872)

1EA30B11-24D3-50DC-812A-FDF10A80B5BF

##### Notes

Last collected on the Peaks in 1934

#### Protandrena (Pterosarus) atricornis

(Cresson, 1878)

F579F21A-1A0C-552D-B793-634C0467A70C

##### Notes

Last collected on the Peaks in 1934

#### Protandrena (Pterosarus) boylei

(Cockerell, 1896)

13BA9562-77B9-5197-B666-B1F3C054FC0A

##### Notes

Last collected on the Peaks in 1934

#### Protandrena (Pterosarus) illustris

(Timberlake, 1967)

59525654-F81D-55B8-AF97-5B63C060D0BD

##### Notes

Last collected on the Peaks in 1934

#### Protandrena (Pterosarus) porterae

(Cockerell, 1900)

C3E5BA68-B867-534B-AD98-715A29ECFEDD

##### Notes

Last collected on the Peaks in 1934

### Apidae (n = 95)

#### Anthophora (Anthophoroides) californica

(Cresson, 1869)

881812EB-7BB2-5C04-9507-5B42A70E9E66

#### Anthophora (Anthophoroides) marginata

(Smith, 1854)

57C635A3-CF90-54C9-B181-7AA2C19FF86F

##### Notes

Last collected on the Peaks in 1950

#### Anthophora (Clisodon) terminalis

(Cresson, 1869)

6C716627-8356-5614-A048-44D41F3A6FCD

#### Anthophora (Lophanthophora) affabilis

Cresson, 1878

BBFF9861-254C-5843-AC1B-7104CA3287D9

#### Anthophora (Lophanthophora) coptognatha

Timberlake, 1951

1774824C-F765-511F-9156-1967A0D36ABE

#### Anthophora (Lophanthophora) porterae

Cockerell, 1900

231EC23F-EAB4-53EA-A453-1503D2B54C71

#### Anthophora (Lophanthophora) ursina

Cresson, 1869

BB4690FF-4BAB-508A-AFE7-24BFC6D1FF71

#### Anthophora (Micranthophora) exigua

Cresson, 1879

81FBFCCA-3DB9-5745-85D3-E25E1C7DA2EA

#### Anthophora (Micranthophora) mortuaria

Timberlake, 1937

0993D304-79EA-5126-BB2F-F789B1304A33

#### Anthophora (Micranthophora) petrophila

Cockerell, 1905

64CED378-58B9-53EF-985B-6CCBA9D0D55D

#### Anthophora (Mystacanthophora) montana

(Cresson, 1869)

ED9C59A1-A894-5117-95EE-4E8BEBA156CC

#### Anthophora (Mystacanthophora) urbana

(Cresson, 1878)

09E57354-58E3-5150-AB6A-1BF1091800DF

#### Anthophora (Pyganthophora) lesquerellae

(Cockerell, 1896)

85002FA7-FCD2-595E-912C-AF6C54A7744F

#### Anthophora (Pyganthophora) vannigera

Timberlake, 1951

8F15967C-4BF3-58F2-B8E0-6E4A33C0BAE6

#### Apis (Apis) mellifera

Linnaeus, 1758

AD05F484-EB86-5DF2-A3E9-035AD77BECE4

#### Bombus (Bombias) nevadensis

Cresson, 1874

69BE6BA7-1C79-5C68-8FD8-D85E93B968E9

#### Bombus (Bombus) occidentalis

Greene, 1858

97480866-BB49-5C87-8C41-1185424A51C3

#### Bombus (Cullumanobombus) morrisoni

Cresson, 1878

BB8FF8B3-5928-582A-9602-C11A43A1DB56

#### Bombus (Cullumanobombus) rufocinctus

(Cresson, 1863)

61002286-D32C-5673-B728-E30210ECB5D7

#### Bombus (Psithyrus) insularis

(Smith, 1861)

C5BAB641-7876-5B79-B54F-B08B5A39F10A

#### Bombus (Psithyrus) variabilis

(Cresson, 1872)

27B4DBCD-6EBE-5B6A-B840-F88C3E85D691

##### Notes

Last collected on the Peaks in 1934

#### Bombus (Pyrobombus) bifarius

Cresson, 1878

19CF1D60-686C-5314-9F82-AB1E993596AA

#### Bombus (Pyrobombus) centralis

Cresson, 1864

FF42A1CB-F814-5282-B79E-E8E4C6B293F1

#### Bombus (Pyrobombus) flavifrons

Cresson, 1863

843CD184-8F64-5042-9561-D04A8471A5C5

#### Bombus (Pyrobombus) huntii

Greene, 1860

215807B2-E8A0-51C3-8285-033FEC1666BF

#### Bombus (Pyrobombus) melanopygus

Nylander, 1848

B855CDCC-AB96-57BB-9267-B18AA5963101

#### Bombus (Pyrobombus) sylvicola

Kirby, 1837

ACDF91D2-2723-56E7-88AC-1B4583074676

##### Distribution

Our record is the first documentation of this species in northern Arizona. Species occurs in neighboring areas.

#### Bombus (Subterraneobombus) appositus

Cresson, 1878

D985F9EF-BB41-59EB-B16D-C2761C86F2BE

#### Bombus (Thoracobombus) californicus

Smith 1854

BE798AAC-77C0-5027-A225-9291AAA655C7

#### Bombus (Thoracobombus) fervidus

(Fabricius, 1798)

DDD82638-7E4F-5449-BC21-177FC6D37871

#### Centris (Paracentris) rhodopus

Cockerell, 1897

3C816D2B-A855-517D-B9D1-B2ACE5F910F3

##### Notes

Last collected on the Peaks in 1936

#### Ceratina (Ceratinula) arizonensis

Cockerell, 1898

56DB7CEB-494C-5DDD-A475-7C13B04E0797

#### Ceratina (Zadontomerus) apacheorum

Daly, 1973

3F3B35EE-A673-557F-9B24-44D4361F1800

#### Ceratina (Zadontomerus) nanula

Cockerell, 1897

5A22010D-B8B0-505C-AAE7-ACEA87698113

#### Ceratina (Zadontomerus) neomexicana

Cockerell, 1901

8D28E922-E1D5-5F3B-B1B8-C17EAE7174F2

#### Ceratina (Zadontomerus) pacifica

H.S. Smith, 1907

198583E5-7DEB-5303-A8C0-B772E7375990

#### Ceratina 001

Latreille, 1802

7CEC19A9-8C9F-5700-AAEA-08293162C8BA

#### Diadasia (Coquillettapis) australis

(Cresson, 1878)

9421FB6C-7C0E-5D32-9759-FE7B6413CE08

#### Diadasia (Coquillettapis) diminuta

(Cresson, 1878)

8A1F6FE9-F8E8-5C70-9DD6-C0CD0F9E58F7

#### Diadasia (Coquillettapis) enavata

(Patton)

2249A62A-2664-520E-BFDB-91843641A887

#### Diadasia (Coquillettapis) rinconis

Cockerell, 1897

E3E69466-60F7-5F86-8F58-B32379C018ED

#### Diadasia (Dasiapis) ochracea

(Cockerell, 1903)

9C20E895-676A-5ABF-9A60-123F1D1584CE

#### Epeolus compactus

Cresson, 1878

01911AC1-9778-540E-9551-D9E2F59C9381

#### Epeolus flavofasciatus

(Smith, 1879)

4C562B3C-7A54-5487-B63A-C07F13BD1B63

##### Notes

Last collected on the Peaks in 1961

#### Epeolus interruptus

Robertson, 1900

56B652D0-78A0-5539-BE2A-8115569BB02B

#### Epeolus pusillus

Cresson, 1864

4F4054C0-185B-58AC-A352-939D8A501110

#### Ericrocis lata

(Cresson, 1878)

D65AA518-D59D-5D34-9300-7526D032B7E0

##### Notes

Last collected on the Peaks in 1936

#### Eucera (Synhalonia) fulvitarsis annae

(Cresson, 1878)

3D7D2BBA-6C95-5A54-9B55-F68E07E1E41B

##### Distribution

Our records are the first documentation of this species in Arizona. Species occurs in neighboring areas.

#### Eucera (Synhalonia) lutziana

(Cockerell, 1933)

1DE722EF-E89E-5133-A129-CECCD75522F0

##### Distribution

Our records are the first documentation of this species in Arizona and the southernmost extension of its range. Species occurs in neighboring areas.

#### Eucera (Synhalonia) primiveris

(Timberlake, 1969)

1451C664-8283-5895-AA5F-2E7D47A4B821

#### Eucera (Synhalonia) speciosa

(Cresson, 1878)

7BBBBCFA-A70D-564A-B0B7-F282476556D2

##### Distribution

Our records are the first documentation of this species in Arizona and the southernmost extension of its range. Species occurs in neighboring areas.

#### Eucera (Synhalonia) territella

(Cockerell, 1905)

77973694-8E46-5163-82EB-F1B23BBBC93F

#### Eucera (Synhalonia) 001

Patton

5064B76A-3934-57E0-8BC3-62A66846AD5F

#### Eucera (Tetraloniella) crenulaticornis

(Cockerell, 1898)

3B719BF3-D987-5373-8118-A4BE188205DE

#### Eucera (Tetraloniella) lippiae

(Cockerell, 1904)

07B13FE4-7890-5063-A1BC-BD9B59F48D02

##### Notes

Last collected on the Peaks in 1934

#### Eucera (Tetraloniella) ochraea

(LaBerge, 2001)

CF0AE348-1E9E-5354-84B4-61517A87961A

##### Notes

Last collected on the Peaks in 1952

#### Eucera 001

Scopoli, 1770

6B47BF03-61A4-5D4F-8E25-E65ACD53122B

#### Eucera 002

Scopoli, 1770

4D91EE8B-D621-5268-84E8-CA09479A6EDD

#### Eucera 003

Scopoli, 1770

90276146-B226-5EFF-9945-7932D8309A1F

#### Exomalopsis (Stilbomalopsis) solani

Cockerell, 1896

975FD7ED-63E7-5D03-A4D8-903B79585AEB

#### Exomalopsis (Stilbomalopsis) solidaginis

Cockerell, 1898

B62F14B8-F410-598D-BFFF-1E2682EEDD0C

#### Holcopasites stevensi

(Crawford, 1915)

83D3B5F6-1C41-5E2E-8D1D-C75499900DCD

#### Melecta (Melecta) bohartorum

Linsley, 1939

77C50B6B-14C0-578E-9DC9-41C53A377840

#### Melecta (Melecta) pacifica

(Cresson, 1878)

09E87481-7C7E-561F-8653-B337F73D9259

#### Melissodes (Callimelissodes) glenwoodensis

Cockerell, 1905

07C8BD2B-5184-562B-B2CB-0B1CADA85496

#### Melissodes (Eumelissodes) confusus

Cresson, 1878

62C38DEA-3206-5D55-86D4-D9EB89D62387

#### Melissodes (Eumelissodes) druriellus

(Kirby, 1802)

538577C4-918F-5C49-9FFE-2ED001C8692B

#### Melissodes (Eumelissodes) fasciatellus

LaBerge, 1961

31F704B2-DBE8-5D62-A316-D51B6F8B8149

#### Melissodes (Eumelissodes) montanus

(Cresson, 1878)

6DA62249-6A10-51B1-B95E-A6C05F05B8FF

#### Melissodes (Eumelissodes) pallidisignatus

Cockerell, 1905

02CECEEF-BE99-53BF-B834-3B8927A608DC

#### Melissodes (Eumelissodes) perpolitus

LaBerge, 1961

A8A65DC4-5EBD-5FA9-A5AA-2D512008E498

#### Melissodes (Eumelissodes) saponellus

Cockerell, 1908

A1BC510E-B1A8-52E5-804E-49D5C263BFBF

##### Distribution

Our records are the first documentation of this species in northern Arizona. Species occurs in neighboring areas.

#### Melissodes (Eumelissodes) semilupinus

Cockerell,1905

365E6DF7-1BDC-5ABC-A421-02319C597693

#### Melissodes (Eumelissodes) tristis

Cockerell, 1894

423D4BFA-89C0-5371-BD4E-26CA0EDFDBDF

#### Melissodes (Eumelissodes) verbesinarum

Cockerell, 1905

FD8E71F3-2B71-5F24-ADC7-5AE6069BA948

#### Melissodes (Heliomelissodes) rivalis

Cresson, 1872

13550B29-21F9-5E7A-BD88-791ABFB26FFF

#### Melissodes (Melissodes) communis

Cresson, 1878

94B687AF-53B9-5F2A-B225-0358B1545C69

#### Melissodes (Melissodes) gilensis

Cockerell, 1896

DE431962-A538-5F9A-9D91-DA9B394760EF

#### Melissodes (Callimelissodes) coloradensis

Cresson, 1878

11AC8B2B-59D1-581E-873D-50E2652D6F18

##### Notes

Last collected on the Peaks in 1938

#### Melissodes (Callimelissodes) compositus

Tucker, 1909

DE709306-02C6-52AF-BDD4-FABC1A2E0786

##### Notes

Last collected on the Peaks in 1950

#### Melissodes (Eumelissodes) agilis

Cresson, 1878

719DC6A9-024E-5245-810E-47A7074090DE

##### Notes

Last collected on the Peaks in 1966

#### Melissodes (Eumelissodes) bimatris

LaBerge, 1961

CEE5160B-0247-5905-913E-30278C796640

##### Notes

Last collected on the Peaks in 2002

#### Melissodes (Eumelissodes) coreopsis

Robertson, 1905

2A4A4B8C-4472-5513-AC92-9AEA2918EF99

##### Notes

Last collected on the Peaks in 1934

#### Melissodes (Eumelissodes) grindeliae

Cockerell, 1898

BAEE7A38-FB9E-5CF0-9077-DDA4AB033909

##### Notes

Last collected on the Peaks in 1964

#### Melissodes (Eumelissodes) menuachus

Cresson, 1868

F5B95FE5-509E-5EC8-BBBD-64F766F4C036

##### Notes

Last collected on the Peaks in 1939

#### Melissodes (Melissodes) paroselae

Cockerell,1905

F59C46D8-30FF-5431-AE0E-2C8297384434

##### Notes

Last collected on the Peaks in 1936

#### Nomada texana

(Cresson, 1872)

53CC35DE-C07A-5EAD-A7DE-9B7DFA517DC4

##### Notes

Last collected on the Peaks in 1952

#### Nomada utahensis

Moalif, 1988

67B88550-DE12-5065-8C0F-87146F814D08

##### Notes

Last collected on the Peaks in 1951

#### Nomada zebrata

Cresson, 1878

4679D53A-6335-52C6-A36B-1A83E1C64E55

##### Notes

Last collected on the Peaks in 1955

#### Svastra (Epimelissodes) obliqua

(Say, 1837)

9670619C-F80F-5F25-96A5-DC19B8E93599

#### Triepeolus 001

Robertson, 1901

4D740556-8DCE-589A-B4DF-B45C2A6FB8ED

#### Triepeolus 003

Robertson, 1901

9F0ACE0A-A3C4-52C1-8504-12F3D581D68D

#### Triepeolus rhododontus

Cockerell, 1921

574308C0-060C-50D7-A408-12AA7B168E40

#### Xeromelecta (Melectomorpha) californica

(Cresson, 1878)

7CF1B193-1F5A-5A78-BB24-2DB58B84A81B

#### Xylocopa (Xylocopoides) californica

Cresson, 1864

A3779B64-DDE3-5E2F-9EAD-856F3271974F

### Colletidae (n = 21)

#### Colletes bryanti

Timberlake, 1951

89A18588-76FD-5387-BCBA-E0D2DE6C7A11

#### Colletes compactus

Cresson, 1868

095260D2-E700-5B46-B10B-FC4A4535D13A

#### Colletes eulophi

Robertson, 1891

99466871-F606-5596-AB78-E2594FA12E6F

##### Notes

Last collected on the Peaks in 1952

#### Colletes gilensis

Cockerell, 1897

73436295-6217-524B-883A-B4E46002BEC5

#### Colletes kincaidii

Cockerell, 1898

EF41FE6E-DAAE-5F20-BC94-D6258023DFEF

#### Colletes paniscus paniscus

Viereck, 1903

52831829-81C4-577B-856F-F98A76B3AD12

#### Colletes scopiventer

Swenk, 1908

6A901250-C3FA-53C8-9710-A6304661DCE7

#### Colletes simulans

Cresson, 1868

363F7B1F-E1D6-5A26-9544-86AA505611FB

##### Notes

Last collected on the Peaks in 1939

#### Colletes wickhami

Timberlake, 1943

E6353080-AD56-59BB-9D55-0F1B73D155C5

#### Colletes wootoni

Cockerell, 1897

CF5525AE-0A6E-5924-BA68-2C396C09C39B

##### Notes

Last collected on the Peaks in 1950

#### Colletes 001

Latreille, 1802

19D320CB-40A0-50C1-9968-F5ECB5CF68F0

#### Colletes 002

Latreille, 1802

309A114F-900F-5C95-90E7-0BBD0FCDAE03

#### Colletes 003

Latreille, 1802

0FA55442-D987-5009-B1B2-DFADE29E0F17

#### Colletes 004

Latreille, 1802

2D8C9376-57BB-557B-8C68-8ACD253CDC1E

#### Colletes 005

Latreille, 1802

F3C82C88-4447-5B3F-A2A5-7862A09D7152

#### Hylaeus (Hylaeus) annulatus

(Linnaeus, 1758)

54ACD16F-8F03-526F-B6DF-A5912CAACD3E

#### Hylaeus (Hylaeus) rudbeckiae

(Cockerell and Casad, 1895)

3D6BC026-4750-53C0-97BB-BDAB456BB5B3

#### Hylaeus (Paraprosopis) cookii

(Metz, 1911)

3A6E9F36-C2B9-56F2-A436-E8E294FAC256

#### Hylaeus (Paraprosopis) wootoni

(Cockerell, 1896)

9EF85B97-E096-5380-8C4D-F097AF97D68F

#### Hylaeus (Prosopis) episcopalis episcopalis

(Cockerell, 1896)

C4CFBB53-8F71-57D6-A367-1949EB65E74D

#### Hylaeus (Prosopis) insolitus

Snelling, 1966

8D54DDCC-DAB2-5C38-8B03-7D172B0286AA

##### Notes

Last collected on the Peaks in 1950

### Halictidae (n = 45)

#### Agapostemon (Agapostemon) angelicus

Cockerell, 1924

A61A09B3-8018-5011-A577-65F1D780E0FD

#### Agapostemon (Agapostemon) melliventris

(Cresson, 1874)

55381D3E-CA54-509E-AC82-601E4B1EC4DE

#### Agapostemon (Agapostemon) texanus

Cresson, 1872

F081C65F-8D69-5BC0-A3F6-562FBABF67EB

#### Dieunomia (Dieunomia) apacha

Cresson, 1868

EB5F8CEA-152E-5647-BCEE-3AEE21FAD544

#### Dieunomia (Epinomia) micheneri

(Cross, 1958)

9A508195-04C3-5F5B-8F0D-E74AC60A8AF8

#### Dieunomia (Epinomia) nevadensis

(Cresson, 1874)

ACB6F9E6-ECD9-524F-9875-6D3F4C05B247

#### Halictus (Nealictus) farinosus

(Smith, 1853)

BB2C1310-0DC0-523D-9808-24DD3FEC6848

#### Halictus (Odontalictus) ligatus

(Say, 1837)

779E5604-A561-55DE-9C99-95436040049F

#### Halictus (Seladonia) confusus

(Smith, 1853)

51976C15-6CB7-5AFB-818E-8C8947286AFB

##### Distribution

Our record is the first documentation of this species in Arizona. Species occurs in neighboring areas.

#### Halictus (Seladonia) tripartitus

(Cockerell, 1895)

131867BE-FE75-5D0F-B0CD-BF34B50D66D2

#### Lasioglossum (Dialictus) aff. comulum

6B9F643F-C2BB-5843-81F1-1F6D3E15E894

#### Lasioglossum (Dialictus) hudsoniellum

(Cockerell, 1919)

3EA4C936-805F-5776-8DDB-3FC61AEF2D06

#### Lasioglossum (Dialictus) hyalinum

(Crawford, 1907)

6126A584-0A67-575B-A841-A7E3FB28C672

#### Lasioglossum (Dialictus) microlepoides

(Ellis, 1914)

40F66CC3-4640-5ED6-9684-162D328D637C

#### Lasioglossum (Dialictus) obnubilum

(Sandhouse, 1924)

61E3D8F6-9F67-5266-BA2F-0DFE16AE48F9

#### Lasioglossum (Dialictus) occidentale

(Crawford, 1902)

FCAD2544-FA92-5432-ADA4-6D88A40450FC

#### Lasioglossum (Dialictus) pallidellum

(Ellis, 1914)

AFEDFE4B-9D7C-59AD-916C-5916D8C1AC97

#### Lasioglossum (Dialictus) cf. perdifficile

0ED293BE-79C2-5CF3-B12F-0037C4347CD4

#### Lasioglossum (Dialictus) aff. perparvum

12C98648-4BE5-5E0B-9B34-05E7A155B7A8

#### Lasioglossum (Dialictus) ruidosense species-group

EC04E4E0-92BE-5D84-A776-37A8A89CC0D5

#### Lasioglossum (Dialictus) semicaeruleum

(Cockerell, 1895)

5E6E032A-0E15-545B-AB7C-CACF11945C97

#### Lasioglossum (Dialictus) new tegulare species-group

B212D413-2750-5BD2-AFE5-76C74F87371F

#### Lasioglossum (Dialictus) cf. viridatulum

BE39684D-D5BE-5690-AF4A-364A2965B659

#### Lasioglossum (Dialictus) 001

3F96DEE1-890A-5B53-A23E-F94615533EEE

#### Lasioglossum (Dialictus) 002

A5AB3A91-8526-578A-A285-8CCE0DD8F745

#### Lasioglossum (Dialictus) 003

E6496AF4-65AD-58B4-9072-508A299027FE

#### Lasioglossum (Dialictus) 004

CF1D1E27-A717-5C25-A06F-FAABDD9487A9

#### Lasioglossum (Dialictus) 005

AF4B7621-C065-5139-9BCB-87E347C6BE9E

#### Lasioglossum (Dialictus) 006

9CCFA909-7A6F-5596-94E8-8EC89A729073

#### Lasioglossum (Dialictus) 007

CFA20CB8-BD7D-58CE-B619-515AA8B7BA73

#### Lasioglossum 008

Curtis, 1833

A123E3A3-F5B3-59FD-AC90-4426D333A7BD

#### Lasioglossum (Hemihalictus) ruficorne

(Crawford, 1907)

A91D4F2E-C135-5FBD-A4DE-2EC674350ED9

#### Lasioglossum (Lasioglossum) desertum

(Smith, 1879)

6C505270-36E5-566E-BA24-F5317EDF4882

#### Lasioglossum (Lasioglossum) egregium

(Vachal, 1904)

B600CEED-6991-5089-BC28-2F2232BA2985

#### Lasioglossum (Lasioglossum) sisymbrii

(Cockerell, 1895)

BB4F1AB0-40AD-5DCB-82AE-E7AEC40F6A5C

#### Lasioglossum (Lasioglossum) trizonatum

(Cresson, 1874)

8D08A081-A556-5290-A02D-47EC4105EB20

#### Lasioglossum (Sphecodogastra) boreale

(Svensson, Ebmer and Sakagami, 1977)

C8C91C4F-4854-5F9B-8ECE-15E2739594FD

#### Nomia (Acunomia) foxii

Dalla Torre, 1896

E420AEB6-29EF-5086-BA48-1920649D519B

##### Notes

Last collected on the Peaks in 1952

#### Nomia (Acunomia) tetrazonata

Cockerell, 1910

377F7BBE-C739-5274-B365-ED5BB0A36625

##### Notes

Last collected on the Peaks in 1936

#### Protodufourea eickworti

Bohart and Griswold, 1997

1C66F359-655F-5550-AA0D-C37E8CD6413B

#### Sphecodes pecosensis

(Cockerell, 1904)

020AC94A-577D-5276-B5ED-EFA3012A8218

#### Sphecodes 001

Latreille, 1804

C02DE143-777D-50F0-903D-12FAF3776917

#### Sphecodes 002

Latreille, 1804

BB499844-23A4-538C-9A10-F0312628F90C

#### Sphecodes 003

Latreille, 1804

C2222EDD-CAE1-54AF-BA73-4CB82F865207

#### Sphecodes 004

Latreille, 1804

B9CF8DEF-302F-5610-801E-C234003C0C46

### Megachilidae (n = 126)

#### Anthidiellum (Loyolanthidium) notatum

Latreille, 1809

BAE6A4CA-C990-570A-93D0-44A8554A33E9

##### Notes

Last collected on the Peaks in 1986

#### Anthidium (Anthidium) atripes

Cresson, 1879

A53509B1-34B9-5A11-9CC1-0AAF642445E0

#### Anthidium (Anthidium) clypeodentatum

Swenk, 1914

3C4145DC-12A1-5CC9-B4AF-38E641DB88B2

#### Anthidium (Anthidium) cockerelli

Schwarz, 1928

88C5F24F-058B-5AAA-B070-CECCF1249F0D

#### Anthidium (Anthidium) dammersi

Cockerell, 1937

B5CC4AB9-A72E-50BF-9883-080D5F5897D1

#### Anthidium (Anthidium) duomarginatum

Gonzalez and Griswold, 2013

93B1174A-5EBD-5BE4-BF2B-7DE58671B0F4

#### Anthidium (Anthidium) emarginatum

(Say, 1824)

04D69FB2-D395-5C1D-B82D-1F15A3AF99A1

#### Anthidium (Anthidium) illustre

(Cresson, 1879)

ACAC14C8-EF0B-593C-BA13-12C97EE918F2

#### Anthidium (Anthidium) maculifrons

Smith, 1854

125BAE85-62A9-5F75-A19E-F4943C67F057

#### Anthidium (Anthidium) maculosum

Cresson, 1878

68A36BC9-833C-50D1-AE95-B8056720933C

#### Anthidium (Anthidium) mormonum

Cresson, 1878

62FA6188-87FC-5E04-B993-1756158EEF24

#### Anthidium (Anthidium) palmarum

Cockerell, 1904

0BE6422D-910E-5624-8E0C-AD3E96E16D83

#### Anthidium (Anthidium) porterae

Cockerell, 1900

FDA5046E-05EF-5B8A-A177-1124D76A49FF

#### Anthidium (Anthidium) schwarzi

Gonzalez and Griswold, 2013

03113CE2-58FA-5BF3-87E2-81CE6C718C25

#### Ashmeadiella (Arogochila) timberlakei

Michener, 1936

9CF8445F-BDA8-547C-BD81-8F34035951C8

##### Distribution

Our records are the first documentation of this species in Arizona. Species occurs in neighboring areas.

#### Ashmeadiella (Ashmeadiella) aridula

Cockerell, 1910

C9200FFA-D0C1-5579-A0C1-285750113348

#### Ashmeadiella (Ashmeadiella) bucconis

(Say, 1837)

8B69962F-2C49-50DE-9DAE-66393E8866ED

#### Ashmeadiella (Ashmeadiella) cactorum basalis

(Cockerell, 1897)

A7785634-EC5B-532B-AAE9-AC4809FDED78

#### Ashmeadiella (Ashmeadiella) californica

(Ashmead, 1897)

FA1FDF0A-E5C9-525E-8AE5-5C6B6D8F2299

#### Ashmeadiella (Ashmeadiella) gillettei

Titus, 1904

21124E45-8257-5766-8076-4C766A573895

#### Ashmeadiella (Ashmeadiella) meliloti

(Cockerell, 1897)

7546446C-5771-53AF-94B1-FEF0A6CAC973

#### Ashmeadiella (Ashmeadiella) opuntiae

(Cockerell, 1897)

CE127362-721B-528D-A2C7-1F692299BF80

#### Ashmeadiella (Ashmeadiella) sonora

Michener, 1939

85B8E1EE-C86E-5FF0-A106-D478A5005BF6

#### Ashmeadiella (Ashmeadiella) vandykiella

Michener, 1949

B102C47A-9B80-5CF0-9352-5EC3FD0B7BBD

##### Distribution

Our record is the first documentation of this species in northern Arizona. Species occurs in neighboring areas.

#### Ashmeadiella 002

Michener, 1939

12C4C393-B89A-5BBA-ADC6-3EE608E1DB5E

#### Atoposmia (Eremosmia) enceliae

(Cockerell, 1935)

7C0BAE73-8CAE-5976-9863-9FA3BD8A7580

#### Coelioxys (Boreocoelioxys) moestus

Cresson, 1864

4D72E702-1E52-5293-8583-CA9871F37AC3

#### Coelioxys (Boreocoelioxys) octodentatus

Say, 1824

BB9894C9-A340-5DD8-9605-D2DB2363FAB1

##### Notes

Last collected on the Peaks in 1950

#### Coelioxys (Boreocoelioxys) porterae

Cockerell, 1900

0A4D28B0-FE00-566C-BDB7-02E4F5B47581

##### Notes

Last collected on the Peaks in 1971

#### Coelioxys (Boreocoelioxys) rufitarsis

Smith, 1854

7679E5FE-BD0E-51CC-9898-5641C9DBD3A4

#### Coelioxys (Coelioxys) sodalis

Cresson, 1878

4BD08A37-08A0-5B43-972E-B773633733B0

##### Notes

Last collected on the Peaks in 1971

#### Coelioxys (Cyrtocoelioxys) gilensis

Cockerell, 1898

00F26440-024B-5D85-A354-2F31B746D29F

#### Coelioxys (Synocoelioxys) apacheorum

Cockerell, 1900

C6FFEE43-6CD3-5816-8F7F-0667F6E4AE4A

##### Notes

Last collected on the Peaks in 1961

#### Coelioxys (Synocoelioxys) erysimi

Cockerell, 1912

BB0A1D90-10A0-5375-8C3A-FD5990CC0325

##### Notes

Last collected on the Peaks in 1971

#### Coelioxys 001

Latrielle, 1809

1C3226BB-605C-5440-B92A-EFA651385ACD

#### Coelioxys 002

Latrielle, 1809

22478A7D-2496-55C3-9BE7-EBF5D7598E18

#### Coelioxys 003

Latrielle, 1809

3DB0592C-A6AB-5C98-97D6-3C5732F643AE

#### Dianthidium (Adanthidium) arizonicum

(Rohwer, 1916)

641219AC-313A-531A-A03C-960307F35BBC

##### Notes

Last collected on the Peaks in 1967

#### Dianthidium (Adanthidium) texanum

(Cresson, 1878)

749C398C-2064-54D1-A717-E0DDB1267E1E

#### Dianthidium (Dianthidium) concinnum

(Cresson, 1872)

34510EE2-2457-514D-BB22-B0EC56802414

#### Dianthidium (Dianthidium) cressonii

(Dalla Torre, 1896)

16FF1507-3BCB-5F1F-881D-46B401E7C797

#### Dianthidium (Dianthidium) curvatum

(Smith, 1854)

3BDE7590-D51F-5614-84A7-7F3BE2AF024E

#### Dianthidium (Dianthidium) desertorum

Timberlake, 1943

59913C69-A703-5B3B-AA07-8BAD43EDCB93

#### Dianthidium (Dianthidium) heterulkei fraternum

Schwarz, 1940

01BFA83D-8AFB-5506-9057-65816C2A5960

#### Dianthidium (Dianthidium) parvum parvum

(Cresson, 1878)

3D666D51-0826-5D19-9A5D-F298E5A442CC

#### Dianthidium (Dianthidium) platyurum

Cockerell, 1923

C14A7BBC-CD8F-5229-9ADB-23868158000C

#### Dianthidium (Dianthidium) pudicum

(Cresson, 1879)

C2862546-7B1B-57CC-88B8-4594587C8AEB

#### Dianthidium (Dianthidium) singulare

(Cresson, 1879)

178C47DF-663B-5509-A200-658FCB63684D

#### Dianthidium (Dianthidium) subparvum

Swenk, 1914

92AB344E-1C0D-5004-8837-D0F4A97BCB16

#### Dianthidium (Dianthidium) ulkei

(Cresson, 1878)

A2B8AD88-A359-59B3-BF44-E681CBE84E2B

#### Heriades (Neotrypetes) cressoni

Michener, 1938

37047D7D-09E9-5EF8-AEF3-E0810D125318

#### Heriades (Neotrypetes) gracilior

Cockerell, 1897

B1234A4A-CFA6-5858-BEB0-CE4CA20B64C7

#### Heriades (Neotrypetes) micropthalma

Michener, 1954

5EBCD303-52B6-5C46-9BFA-5DD6CDD7F13F

##### Notes

Last collected on the Peaks in 1934

#### Heriades (Neotrypetes) texana

Michener, 1938

C7337709-9CB4-5D9E-88A6-05708BF1758E

##### Notes

Last collected on the Peaks in 1947

#### Heriades (Neotrypetes) timberlakei

Michener, 1938

D00098F2-786C-539F-B1B8-169EDA4EE637

#### Heriades 002

Spinola, 1808

C72F32D2-0B53-5708-9712-FC1C0863C1EC

#### Hoplitis (Alcidamea) grinnelli

(Cockerell, 1910)

53435A45-9C43-5AB9-9A96-EAA39425EEBF

##### Notes

Last collected on the Peaks in 1986

#### Hoplitis (Alcidamea) paroselae

Michener, 1947

749ECFED-338B-5C0F-8D82-B82D404B65F3

#### Hoplitis (Alcidamea) truncata mescalerium

(Cresson, 1878)

0578FF96-A121-5C09-ADF8-C93602AA8BF8

##### Notes

Last collected on the Peaks in 1961

#### Hoplitis (Proteriades) zuni

(Parker, 1977)

DF505F9C-E4C7-5176-A9F4-54AC1D1CED57

##### Distribution

Our records are the first documentation of this species in northern Arizona. Species occurs in neighboring areas.

#### Hoplitis 001

Klug, 1916

FD64131E-8E42-5123-8486-08573F841B9B

#### Lithurgopsis apicalis

(Cresson, 1875)

2156F194-8D81-5D6E-9201-F354CEE9FFAD

#### Lithurgopsis planifrons

(Friese, 1908)

F3A31A76-2213-5CC4-935A-BB5CCAC8B79A

#### Megachile (Argyropile) sabinensis

Mitchell, 1934

179F0CFA-95FC-5BC0-A359-BD62177D10AD

#### Megachile (Chelostomoides) angelarum

(Cockerell, 1902)

3C43EC5B-F658-57F0-BC4F-46751F8866EF

#### Megachile (Chelostomoides) chilopsidis

(Cockerell, 1900)

CF0C95BD-28E7-527A-96FC-193F40622701

#### Megachile (Chelostomoides) lobatifrons

(Cockerell, 1924)

5342E8CB-C6A2-5DBD-8D0D-9C565120D9DE

#### Megachile (Chelostomoides) subexilis

(Cockerell, 1908)

8730FAF1-ABCE-589B-BE68-E9581454F768

#### Megachile (Litomegachile) brevis

Say, 1837

CA4F5EE1-4A73-547D-98C5-64415D02861A

##### Notes

Last collected on the Peaks in 1967

#### Megachile (Litomegachile) lippiae

Cockerell, 1900

55414545-4385-55D5-9299-6EE344FD1B5A

#### Megachile (Litomegachile) onobrychidis

Cockerell, 1908

941697B0-CA32-52B5-A0DC-B402200573BE

#### Megachile (Litomegachile) snowi

Mitchell, 1927

D4F4E31E-3156-5ECF-A9AE-58E223EEB0D9

##### Notes

Last collected on the Peaks in 1950

#### Megachile (Litomegachile) texana

Cresson, 1878

2387BFEC-058E-5148-9CF0-42E574E7C2A5

##### Notes

Last collected on the Peaks in 1950

#### Megachile (Megachile) lapponica

Thomson 1872

9FA9D24F-6A7D-5D55-A4D5-D1EC1072427D

##### Distribution

Our records are the first documentation of this species in Arizona and the southernmost extension of its range. Species occurs in neighboring areas.

#### Megachile (Megachile) montivaga

(Cresson, 1878)

2FCA13AB-9A03-5210-A7A6-3000C023CD9A

#### Megachile (Megachile) relativa

Cresson, 1878

2C3B94E6-982A-561F-839E-6E4ACC8BFD7E

#### Megachile (Megachiloides) manifesta

Cresson, 1878

6B59B132-1445-5F2B-9ADA-4FA2FEF799B1

##### Notes

Last collected on the Peaks in 1951

#### Megachile (Megachiloides) mucorosa

Cockerell, 1908

96819045-E65A-5130-876C-577AFB575762

#### Megachile (Megachiloides) sublaurita

Mitchell, 1927

D6CB5597-7329-5DA5-BF45-FD83AA057913

#### Megachile (Phaenosarus) agustini

Cockerell, 1905

5AAEAB31-8BFD-55D0-94A5-A86929783648

#### Megachile (Phaenosarus) fortis

Cresson, 1872

C61B21B7-4B75-5933-BE5F-FD2F8DA36AB7

#### Megachile (Pseudocentron) sidalceae

Cockerell, 1897

5C144971-E02C-57E1-B0A5-6C6F8B10E945

#### Megachile (Sayapis) fidelis

Cresson, 1878

66BE7DC1-2EDA-5C7A-8AC3-81B53830936D

#### Megachile (Sayapis) inimica sayi

Cresson, 1872

AF20374E-5A07-5254-BA36-0B6611F19F86

#### Megachile (Sayapis) mellitarsis

Cresson, 1878

DF589A16-C83F-5DFB-BE2E-464600EBA21F

#### Megachile (Sayapis) policaris

Say, 1831

1E259CB3-F534-52FB-9C34-09F5E0EF97FB

#### Megachile (Sayapis) pugnata pomonae

Say, 1837

212CB3E5-B659-5307-A02C-36BC70D69FF5

#### Megachile (Xanthosarus) comata

Cresson, 1872

5EBC9F90-67A2-5634-9393-129081BD135C

#### Megachile (Xanthosarus) frigida

Smith, 1853

2E0F4BA3-06C7-5C10-AD6B-AF61D5F953F3

#### Megachile (Xanthosarus) latimanus

(Say, 1823)

39896233-4AFC-56AA-B96A-40C70881FEA0

##### Distribution

Our records are the first documentation of this species in northern Arizona. Species occurs in neighboring areas.

#### Megachile (Xanthosarus) melanophaea

Smith, 1853

1764E39A-99E4-5389-8EDD-80D88D136266

#### Megachile (Xanthosarus) mucida

Cresson, 1878

0604FF57-8863-5796-9E22-D676E87A8985

#### Megachile (Xanthosarus) perihirta

Cockerell, 1898

38B75DF5-11FF-594C-AF7C-AA530A4D22C4

##### Notes

Last collected on the Peaks in 1990

#### Megachile 001

Latreille

321D89DD-E224-51F5-ABAB-93385134CC91

#### Megachile 002

Latreille

6FD35A59-95EB-5845-967E-2669C5444753

#### Osmia (Cephalosmia) montana

Cresson, 1864

E90B9B6E-38A2-518F-94C0-1A58CCDAEFEB

#### Osmia (Cephalosmia) subaustralis

Cockerell, 1900

A9E9D523-92A3-5440-B2C3-886AC1D80423

#### Osmia (Helicosmia) coloradensis

Cresson, 1878

86860BB2-A34B-51B1-AD48-9243CE96AB38

#### Osmia (Cephalosmia) 001

Sladen, 1916

96246B5E-A63C-5756-BB30-86CFAF0E030B

#### Osmia (Cephalosmia) 002

Sladen, 1916

2A4483DB-CF2F-5CAE-9A3E-983BCC505C69

#### Osmia (Helicosmia) texana

Cresson, 1872

07126906-5E9E-576C-8B4C-E9FE989FC67C

#### Osmia (Melanosmia) albolateralis

Cockerell, 1906

373F363D-1B20-54E1-9E2A-13C6FD0F2B11

##### Notes

Last collected on the Peaks in 1971

#### Osmia (Melanosmia) brevis

Cresson, 1864

33BE49FA-2176-5C4B-8E93-DCB38B86F6F3

#### Osmia (Melanosmia) bucephala

(Cresson, 1864)

25C53F95-FE5E-5E04-B860-989E67A94812

##### Distribution

Our records are the first documentation of this species in Arizona. Species occurs in neighboring areas.

#### Osmia (Melanosmia) densa

Cresson, 1864

61C17325-8EF8-5719-A018-74391E60094C

##### Notes

Last collected on the Peaks in 1971

#### Osmia (Melanosmia) juxta

Cresson, 1864

E555D374-0A3D-5030-889F-7B3FFC09CF51

#### Osmia (Melanosmia) liogastra

Cockerell, 1933

4D10EF63-60D3-5F8E-949B-A01C4924E6D6

#### Osmia (Melanosmia) pentstemonis

Cockerell, 1906

8A438904-9830-5276-A73F-E21A74B5D7EE

##### Notes

Last collected on the Peaks in 1950

#### Osmia (Melanosmia) aff. pentstemonis

BC6961F1-01AB-5590-B17C-5C53BE9273F0

#### Osmia (Melanosmia) simillima

(Smith, 1853)

81B70320-D52E-5394-ABCD-4094129EC875

#### Osmia (Melanosmia) unca

Michener, 1937

CBC3DB17-0FFD-5433-B435-E279AC72B8E0

##### Notes

Last collected on the Peaks in 1964

#### Osmia (Melanosmia) 001

Schmiedeknecht, 1885

E7C3157A-FE80-5D23-B0DF-8574F9B6D2F4

#### Osmia (Melanosmia) 002

Schmiedeknecht, 1885

9C4357D9-1796-5603-8189-05855C9BE9DA

#### Osmia (Osmia) lignaria

(Say, 1837)

2BA137A5-FAEF-5E24-B51F-2F1E50F10D57

##### Notes

Last collected on the Peaks in 1971

#### Osmia 002

Panzer, 1806

3A5AD2B3-49E4-5848-BBE6-80EFFB447658

#### Osmia 003

Panzer, 1806

97B4F1DC-12AE-5C19-8436-9876D63A6D5D

#### Osmia 005

Panzer, 1806

FAA4FEE6-14A5-5A38-8C4D-BC886910D7BF

#### Osmia 006

Panzer, 1806

4DE2B128-4594-5228-B1BA-FBDD00F17F70

#### Osmia 007

Panzer, 1806

C84FD703-DCEB-5006-887B-3482E01BC39A

#### Osmia 009

Panzer, 1806

658A6E39-C2FB-512D-9CC1-640CE7A8FE87

#### Osmia 010

Panzer, 1806

15C80295-02F9-5D8E-8AF0-28BABDEFC05D

#### Osmia 011

Panzer, 1806

A8EEB544-FC55-57FA-B343-3F5A69609100

#### Osmia 012

Panzer, 1806

0A77F222-76E2-5D28-A33E-8C015AAFD1E0

#### Paranthidium (Paranthidium) jugatorium

(Say, 1824)

3C5FA2D8-F26D-5421-95DD-7B81A08B3056

#### Stelis (Dolichostelis) rudbeckiarum

(Cockerell, 1904)

036E2376-060D-5AD5-BCC0-C7188E7980A9

#### Stelis (Stelis) elegans

(Cresson, 1864)

C58148E8-6B1D-585B-B304-CF4FB5E56192

## Discussion

**Figure 2.**
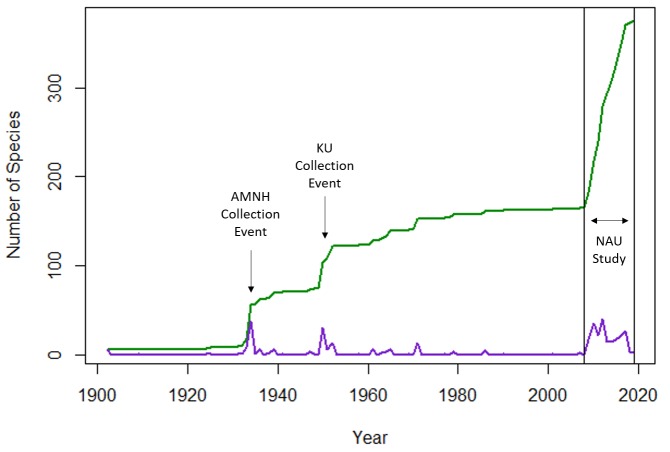
Collection events of new species documentation on the San Francisco Peaks. The purple line represents the number of new species records, and the green line shows the cumulative addition of species to the San Francisco Peaks area.

Prior to the start of our study, past collection events had documented 155 bees on the Peaks between 1908 and 2009. Records that were not identified to species, outside of our collection, were not considered in our checklist. Two collection events in particular significantly advanced the known number of bee species on the San Francisco Peaks (Fig. 2). In 1934, the American Museum of Natural History (Collector E. L. Bell) added 38 new bee species records to the Peaks and in 1950-1952, the University of Kansas (multiple collectors) added 45 new species records. However, of these 155 previously documented records, 68 of them have not been documented since 1971 and 49 of these 68 species were rare and only had one previously documented record. *Perditazebrata and Coelioxysporterae* are of note because they were not collected in our 10-year study but were collected in high abundance in previous years. *P.zebratahad* 150+ records documented in the pinyon-juniper life zone but has not been recorded on the Peaks since 1971. *C.porterae* had 100+ records documented throughout the Peaks since 1952 but was not collected in our 10-year study.

**Figure 3.**
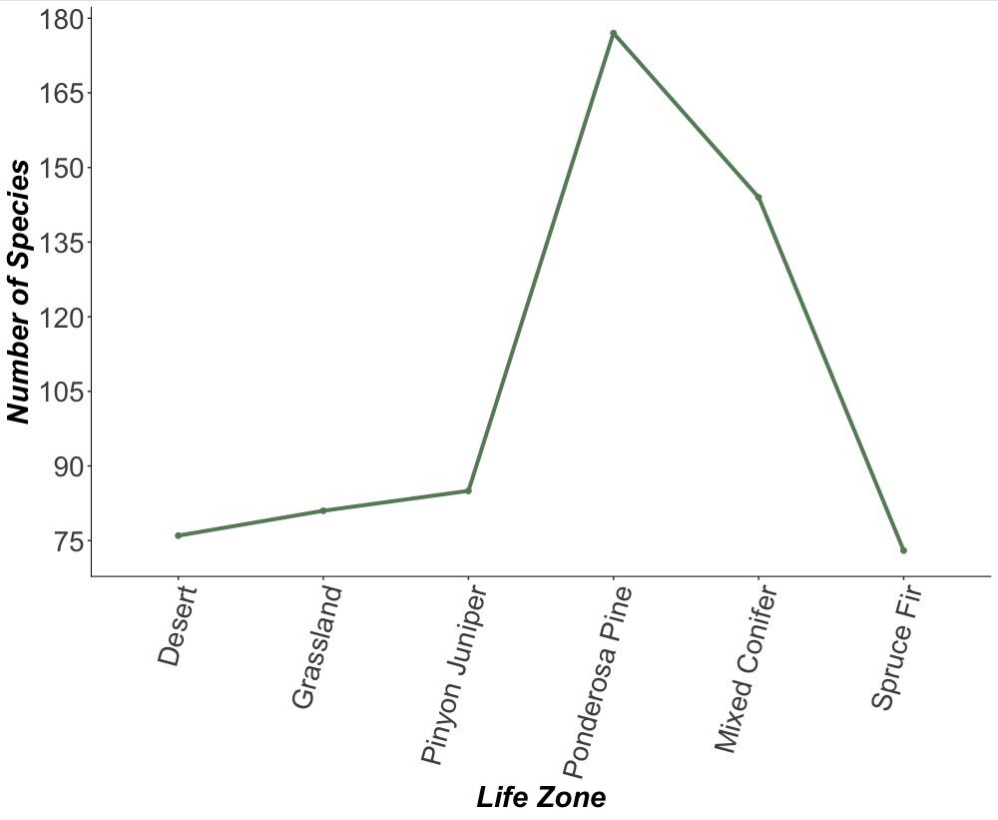
Number of bee species found at each life zone (n=339 species for which we had accurate locality data to assign life zone designations).

Our study that began in 2009 was a collaborative inventory project with Northern Arizona University and United States Fish and Wildlife Service and was started with the intention of documenting all bee species inhabiting the Peaks region. Over a 10-year period, we documented an additional 204 bee species inhabiting the Peaks, leading to a total of 359 bee species recorded on this checklist (7,952 specimens Table 2). The ponderosa pine life zone is the most diverse, with a total of 177 species inhabiting that vegetation zone (Fig. 3). However, collection effort in the desert shrub and desert grassland life zones was disproportionately lower than that of the four higher life zones. Desert shrub and desert grassland were only sampled in 2009-2012. Further, only pollinator cups were used at desert shrub and desert grassland, whereas bug vacuums became the primary method of sampling at the pinyon-juniper, ponderosa pine, mixed conifer, and spruce-fir life zones from 2016-2019. These two components could potentially lead to a higher abundance of specimens collected at those four higher life zones.

Abundances varied between families but generally followed species richness trends. However, 68 species of the 204 newly documented species were singletons and 16 species were highly abundant (averaging over 50 specimens per year) with *Lasioglossumdesertum* being the most abundant species on the Peaks. There were other notable species that were also relatively abundant in specific life zones, such as *Bombusoccidentalis*, which averaged 25 specimens per year in all life zones above ponderosa pine. Of the 204 newly documented species, 15 of these exhibited a range expansion.

**Figure 4.**
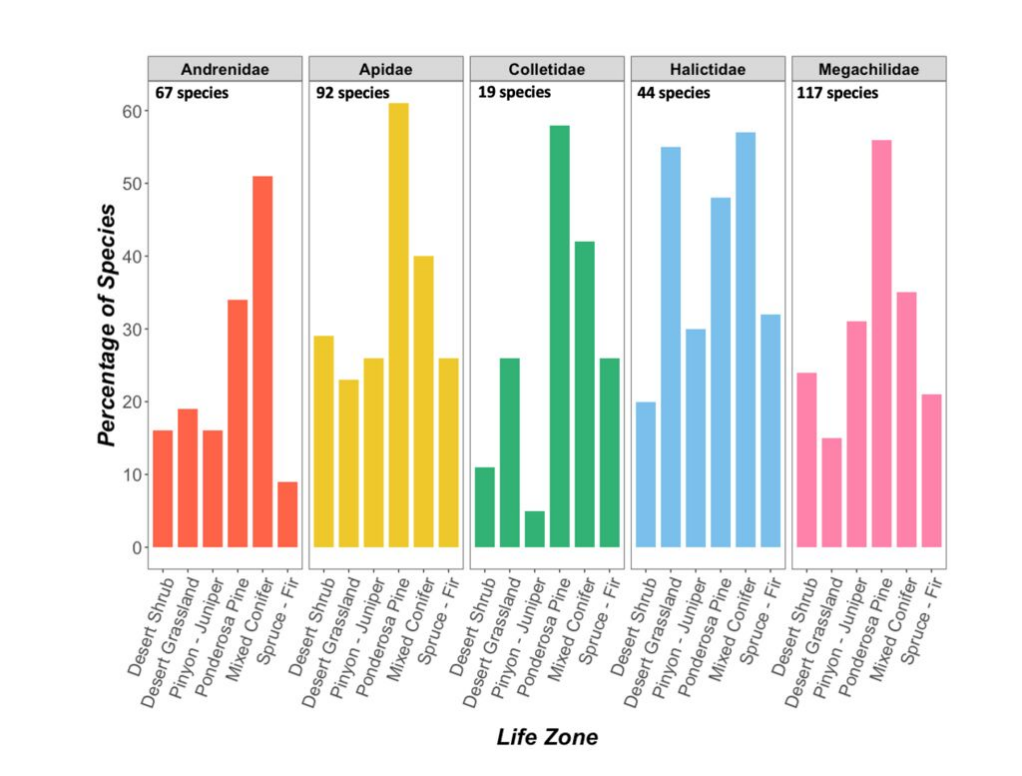
Percentage of species in a family found at each life zone (n=339 species for which we had accurate locality data to assign life zone designations). Numbers for each family sum higher than 100% because of species that occurred in more than one life zone.

**Figure 5.**
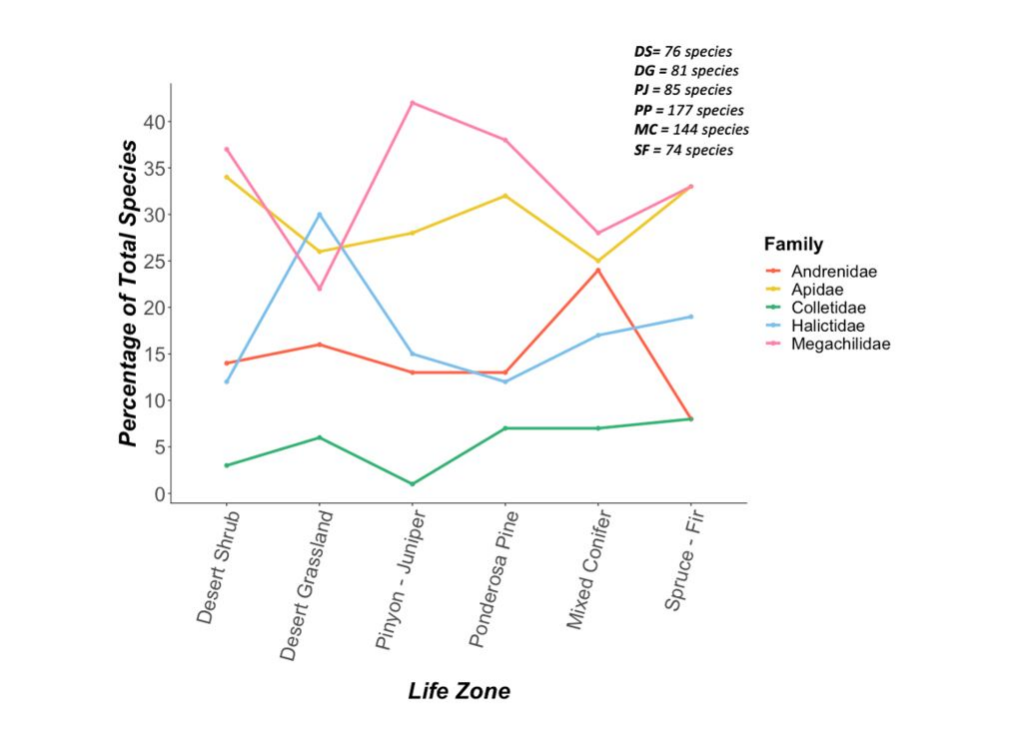
Percent of total species organized by family for each life zone (n=339 species for which we had accurate locality data to assign life zone designations). DS = desert shrub, DG = desert grassland, PJ = pinyon-juniper, PP = ponderosa pine, MC = mixed conifer, SF = spruce-fir. Numbers for each life zone sum to 100%.

All bee families were represented at each one of the life zones (Fig. 4). Megachilids were the most diverse family and they had the highest species richness at ponderosa pine (56% of total megachilid species). This high diversity of megachilids may be explained by an abundance of dead-and-down wood required for nesting by many Megachilini and Osmiini (Sheffield et al. 2011, Cane et al. 2014) which may be restricted to higher environments (McCabe et al. 2019a). Thirty-eight percent of all bee species collected in ponderosa pine are megachilids, further supporting the idea that the woody ponderosa environment is favorable for this diverse bee family (Fig. 5). However, it is also possible that megachilid species may be more common at ponderosa pine simply due to the sheer number of Megachildae inhabiting the Peaks. Apidae is the second most diverse family on our elevation gradient and contributed to a large portion of the species at each life zone. Combined, Megachilidae and Apidae comprise about 60% of the species on the Peaks.

In general, the percentage of species composed by a particular family at each life zone seemed to positively correlate with the overall diversity of that family found on the Peaks. For example, Megachilidae was the most dominant family at each life zone (except for desert grassland), and they were also the most species rich family in the Peaks region. An exception is at the mixed conifer life zone where Andrenidae was the most dominant family (comprised 24% of all species). Further, all but one of our reported *Andrena* species were found to inhabit mixed conifer, and 41% of Andrenidae *only* occurred at mixed conifer. Andrenids are typically ground nesters that prefer the sandy soils found at lower elevations (Michener 2000) and such high species richness of Andrenidae at mixed conifer was unanticipated. The highest diversity of halictids (24 species) is seen at desert grassland and there is little diversity in Halictidae at the higher elevations. This trend is consistent with the idea that lower elevation habitats may provide greater nesting resources for halictids due to the warm, dry environment (Michener 2000, Devoto et al. 2005). Colletidae represented a relatively small subset of bees on the Peaks (5%) but do have representative species at every life zone.

**Figure 6.**
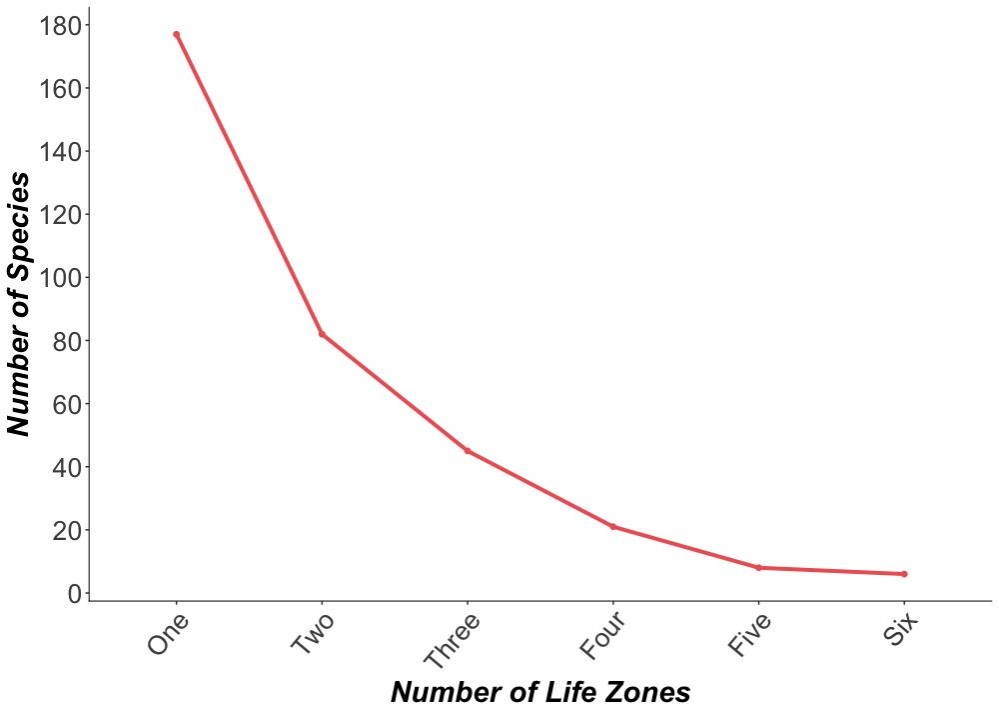
Number of species found to inhabit increasing numbers of life zones. Nearly 50% of total bee species were found in only one life zone (n=339 species for which we had accurate locality data to assign life zone designations).

Our results indicate a high degree of habitat specialization along the elevational gradient of the Peaks, with 49% of the total bee species found in only one life zone (177 species) (Fig. 6). Conversely, only six species (< 1% of the total bee species included in the analysis) have ranges that encompass all six of the life zones included in our study: *Anthophoramontana, Diadasiadiminuta, Melissodestristis, Agapostemonangelicus, Agapostemontexanus*, and *Lasioglossumsisymbri*. These six species only come from two families (Apidae and Halictidae) and all six are ground nesters. Evidence suggests that with changing climate or other anthropogenic disturbances, higher species loss may occur with species that encompass smaller geographic ranges or specialized habitats (Sánchez-Bayo and Wyckhuys 2019). Bees inhabiting higher elevations may be acutely susceptible to climate change; warming temperatures may cause bees to contract upwards in elevation (Hickling et al. 2006). If broad-scale tree reductions continue as predicted (Allen et al. 2015) most megachilids could lose nesting resources. Taxa that reach their highest diversity at higher elevations (e.g. Apidae) are also likely susceptible to climate change. Additionally, lower elevation habitats will get increasingly warmer and are likely to experience more drought events. This could potentially limit the already scarce floral resources available in these desert environments. It is therefore important to document ranges and habitat requirements of the bee species found on the Peaks to predict future shifts in local distribution.

Insect species richness and abundance is reported to be declining globally, pointing to the importance of regularly monitoring populations worldwide (Biesmeijer et al. 2006, Cameron et al. 2011, Bartomeus et al. 2013, Jacobson et al. 2018). However, of the 73 studies summarized by Sánchez-Bayo and Wyckhuys (2019) that indicate a global decline of insects, the majority of the studies that reported bee declines focused mostly on bumble bees. This likened itself to a lack of world records for other native bee species, which this checklist can provide. Ranges for the vast majority of native bee species are still relatively unknown (Bartomeus et al. 2013). Only one species from our checklist, *Bombusoccidentalis*, has published accounts showing population trends (Cameron et al. 2011). Checklists like ours and others (Carril et al. 2018, Parys et al. 2018, Delphia et al. 2019, Stephenson et al. 2018) could serve as important reference points to assess future responses of bees to global change.

## Supplementary Material

85A74899-1530-5E38-A8D4-ACBA285C5782 10.3897/BDJ.8.e49285.suppl1 Supplementary material 1 Localities for 2019 qualitative sampling
Data type decimal latitude/longitude localities File: oo_392852.xlsx https://binary.pensoft.net/file/392852 McCabe et al

F1DB3B69-7782-5FE9-93C7-C22AA707ADDB 10.3897/BDJ.8.e49285.suppl2 Supplementary material 2 Occurrence Records removed from analysis
Data type occurrences File: oo_392854.xlsx https://binary.pensoft.net/file/392854 McCabe et al

CDCE64C2-007D-57EA-BD04-267EF5BA868F 10.3897/BDJ.8.e49285.suppl3 Supplementary material 3 DwC archive file of the bee records on the San Fransico Peaks
Data type occurrences File: oo_362433.csv https://binary.pensoft.net/file/362433 McCabe et al

## Figures and Tables

**Table 1.** List of all 58 NAU sites including latitude, longitude, years sampled and life zone.

**Lifezone**	**Site**	**Years Sampled**	**lat**	**lon**
desert shrub	DS1	2009 - 2012	35.6927	-111.4260
desert grassland	DG1	2009 - 2012	35.5810	-111.6560
pinyon-juniper	PJ1	2016 - 2018	35.4641	-111.5915
pinyon-juniper	PJ2	2016 - 2018	35.4737	-111.5932
pinyon-juniper	PJ3	2016 - 2018	35.4762	-111.6031
pinyon-juniper	PJ4	2016 - 2018	35.4862	-111.5998
pinyon-juniper	PJ5	2016	35.4875	-111.6101
pinyon-juniper	PJ6	2016 - 2018	35.4947	-111.6178
pinyon-juniper	PJ7	2016	35.5138	-111.6237
pinyon-juniper	PJ8	2009 - 2012 & 2016-2018	35.3539	-111.7306
ponderosa pine	PP1A	2013 - 2015	35.3511	-111.7992
ponderosa pine	PP2A	2013 - 2015	35.3453	-111.8041
ponderosa pine	PP3A	2013 - 2015	35.3474	-111.8147
ponderosa pine	PP1	2015 - 2018	35.3857	-111.7367
ponderosa pine	PP2	2015 - 2018	35.4163	-111.6714
ponderosa pine	PP3	2015 - 2018	35.3876	-111.6874
ponderosa pine	PP4	2016 - 2018	35.4270	-111.6963
ponderosa pine	PP5	2009 - 2012 & 2016 - 2019	35.3539	-111.7306
ponderosa pine	PP6	2016 - 2018	35.3889	-111.7251
ponderosa pine	PP7	2016 - 2018	35.3979	-111.7233
ponderosa pine	PP8	2016 - 2018	35.3879	-111.6869
ponderosa pine	PP1F	2013 - 2015	35.3861	-111.7365
ponderosa pine	PP2F	2013 - 2015	35.3897	-111.7245
ponderosa pine	PP3F	2013 - 2015	35.3879	-111.6861
ponderosa pine	Ken1A	2015	35.4263	-111.8199
ponderosa pine	Ken1B	2015	35.4290	-111.8221
ponderosa pine	Ken1C	2015	35.4317	-111.8240
mixed conifer	MC1	2013 - 2019	35.3285	-111.7380
mixed conifer	MC2	2009 - 2018	35.3539	-111.7306
mixed conifer	MC3	2013 - 2018	35.3290	-111.7390
mixed conifer	MC4	2016 - 2018	35.3543	-111.7320
mixed conifer	MC5	2016 - 2019	35.3803	-111.6858
mixed conifer	MC6	2016 - 2018	35.3757	-111.7321
mixed conifer	MC7	2016	35.3790	-111.6942
mixed conifer	MC8	2016	35.3799	-111.6889
mixed conifer	MC1F	2013 - 2015	35.3751	-111.7331
mixed conifer	MC2F	2013 - 2015	35.3798	-111.6847
mixed conifer	MC3F	2013 - 2015	35.3795	-111.6937
mixed conifer	Ken2A	2015	35.4225	-111.8278
mixed conifer	Ken2B	2015	35.4252	-111.8313
mixed conifer	Ken2C	2015	35.4243	-111.8338
spruce-fir	SF1A	2013 - 2015	35.3403	-111.6475
spruce-fir	SF2A	2013 - 2015	35.3386	-111.6506
spruce-fir	SF3A	2013 - 2015	35.3392	-111.6509
spruce-fir	SF1	2015 - 2018	35.3585	-111.7080
spruce-fir	SF2	2015 - 2018	35.3387	-111.6511
spruce-fir	SF3	2015 - 2019	35.3322	-111.6561
spruce-fir	SF4	2016 - 2018	35.3602	-111.7189
spruce-fir	SF5	2016 - 2018	35.3589	-111.7181
spruce-fir	SF6	2016 - 2019	35.3568	-111.7173
spruce-fir	SF7	2016	35.3469	-111.7035
spruce-fir	SF8	2016	35.3463	-111.7066
spruce-fir	SF1F	2013 - 2015	35.3423	-111.6436
spruce-fir	SF2F	2013 - 2015	35.3405	-111.6490
spruce-fir	SF3F	2013 - 2015	35.3373	-111.6529
spruce-fir	Ken3A	2015	35.4149	-111.8361
spruce-fir	Ken3B	2015	35.4167	-111.8389
spruce-fir	Ken3C	2015	35.4194	-111.8396

**Table 2.** Comprehensive list of bee species collected in the San Francisco Peaks region. Each life zone is denoted (DS = desert shrub, DG = desert grassland, PJ = pinyon-juniper, PP = ponderosa pine, MC = mixed conifer, SF = spruce-fir). Notations in the "NAU" column are species that were recorded in the NAU inventory study from 2009–2019. Notations in the "other" (O) column were species recorded to occur on the San Francisco Peaks by other institutions. Notations also designate whether species were collected from cup (C) sampling or flower (F) sampling. Further, rare (R) species (only one specimen collected) are marked with a "*" and abundant (A) species (>100 specimens collected) are marked with an "x".

**Family**	**Genus**	**Species**/ **Morphospsecies**	**Sub**- **species**	**DS**	**DG**	**PJ**	**PP**	**MC**	**SF**	**NAU**	**O**	**C**	**F**	**R**	**A**
Andrenidae	*Andrena*	*algida*			1			1		1		1	1		
Andrenidae	*Andrena*	*amphibola*						1		1		1			
Andrenidae	*Andrena*	*angustitarsata*						1		1		1			
Andrenidae	*Andrena*	*apacheorum*					1	1		1	1	1			
Andrenidae	*Andrena*	*argemonis*					1	1		1	1	1			
Andrenidae	*Andrena*	*auripes*						1		1		1		*	
Andrenidae	*Andrena*	*coconina*									1			*	
Andrenidae	*Andrena*	*commoda*					1	1		1	1	1			
Andrenidae	*Andrena*	*costillensis*						1		1		1			
Andrenidae	*Andrena*	*crataegi*						1		1		1			
Andrenidae	*Andrena*	*cressonii*						1		1		1		*	
Andrenidae	*Andrena*	*crinita*						1		1		1			
Andrenidae	*Andrena*	*cyanophila*			1		1	1	1	1		1	1		x
Andrenidae	*Andrena*	*frigida*					1				1			*	
Andrenidae	*Andrena*	*helianthi*					1			1	1		1		
Andrenidae	*Andrena*	*mariae*						1		1		1		*	
Andrenidae	*Andrena*	*medionitens*						1		1	1	1	1		
Andrenidae	*Andrena*	*micheneriana*			1		1			1		1			
Andrenidae	*Andrena*	*miranda*					1	1		1		1	1		
Andrenidae	*Andrena*	*moquiorum*									1			*	
Andrenidae	*Andrena*	*nubecula*				1	1	1	1	1		1	1		
Andrenidae	*Andrena*	*pecosana*					1			1	1	1		*	
Andrenidae	*Andrena*	*perpunctata*					1	1		1		1		*	
Andrenidae	*Andrena*	*platyrhina*					1			1		1		*	
Andrenidae	*Andrena*	*prunorum*			1	1	1			1		1	1		
Andrenidae	*Andrena*	*simulata*		1						1	1	1			
Andrenidae	*Andrena*	*sonorensis*					1				1			*	
Andrenidae	*Andrena*	*striatifrons*						1		1	1	1		*	
Andrenidae	*Andrena*	*tegularis*							1	1			1	*	
Andrenidae	*Andrena*	*w-scripta*		1				1		1		1			
Andrenidae	*Andrena*	001						1		1		1		*	
Andrenidae	*Andrena*	003						1		1		1		*	
Andrenidae	*Andrena*	004						1		1		1			
Andrenidae	*Andrena*	005						1		1		1		*	
Andrenidae	*Andrena*	006						1		1			1	*	
Andrenidae	*Andrena*	(*Belandrena*) 001			1		1			1		1			
Andrenidae	*Andrena*	(*Diandrena*) 001					1			1		1		*	
Andrenidae	*Andrena*	(*Trachandrena*) 001					1	1		1		1			
Andrenidae	*Calliopsis*	*callops*						1		1		1		*	
Andrenidae	*Calliopsis*	*chlorops*		1	1	1				1	1	1			
Andrenidae	*Calliopsis*	*puellae*		1						1		1			
Andrenidae	*Calliopsis*	*rozeni*									1			*	
Andrenidae	*Calliopsis*	*teucrii*					1				1				
Andrenidae	*Calliopsis*	*timberlakei*			1	1				1		1			
Andrenidae	*Calliopsis*	*zebrata*					1			1	1	1			
Andrenidae	*Calliopsis*	001						1		1		1		*	
Andrenidae	*Macrotera*	*latior*			1	1	1			1	1	1			
Andrenidae	*Perdita*	*giliae*					1				1			*	
Andrenidae	*Perdita*	*gutierreziae*				1					1			*	
Andrenidae	*Perdita*	*sphaeralceae*				1					1				
Andrenidae	*Perdita*	*zebrata*				1					1				x
Andrenidae	*Perdita*	001		1					1	1		1		*	
Andrenidae	*Perdita*	002		1						1		1			
Andrenidae	*Perdita*	003		1	1					1		1			
Andrenidae	*Perdita*	004						1		1		1			
Andrenidae	*Perdita*	005			1					1		1			
Andrenidae	*Perdita*	006			1					1		1			
Andrenidae	*Perdita*	007		1	1					1		1			
Andrenidae	*Perdita*	008		1	1					1		1			
Andrenidae	*Perdita*	009		1						1		1			
Andrenidae	*Perdita*	010		1						1		1			
Andrenidae	*Protandrena*	*albitarsis*									1			*	
Andrenidae	*Protandrena*	*atricornis*							1		1				
Andrenidae	*Protandrena*	*boylei*				1					1			*	
Andrenidae	*Protandrena*	*illustris*									1			*	
Andrenidae	*Protandrena*	*neomexicanus*									1				
Andrenidae	*Protandrena*	*porterae*				1					1			*	
Andrenidae	*Protandrena*	(*Heterosarus*) 001						1		1		1	1		
Andrenidae	*Protandrena*	(*Heterosarus*) 002						1		1		1	1		
Andrenidae	*Protandrena*	(*Heterosarus*) 003						1	1	1			1		
Andrenidae	*Protandrena*	(*Heterosarus*) 004					1	1		1		1	1		
Andrenidae	*Protandrena*	(*Heterosarus*) 005						1		1		1		*	
Apidae	*Anthophora*	*affabilis*		1	1	1	1	1		1		1			
Apidae	*Anthophora*	*californica*						1		1	1	1			
Apidae	*Anthophora*	*coptognatha*		1	1					1		1			
Apidae	*Anthophora*	*exigua*		1	1			1		1		1			
Apidae	*Anthophora*	*lesquerellae*		1		1				1		1			
Apidae	*Anthophora*	*marginata*					1				1				
Apidae	*Anthophora*	*montana*		1	1	1	1	1	1	1	1	1	1		x
Apidae	*Anthophora*	*mortuaria*			1					1		1		*	
Apidae	*Anthophora*	*petrophila*		1	1	1				1		1			
Apidae	*Anthophora*	*porterae*		1	1		1			1		1			
Apidae	*Anthophora*	*terminalis*			1	1	1	1	1	1	1	1	1		x
Apidae	*Anthophora*	*urbana*		1			1	1		1	1	1	1		
Apidae	*Anthophora*	*ursina*					1	1		1		1			
Apidae	*Anthophora*	*vannigera*			1			1		1		1			
Apidae	*Apis*	*mellifera*		1			1	1		1	1	1	1		
Apidae	*Bombus*	*appositus*					1	1		1		1			
Apidae	*Bombus*	*bifarius*					1	1		1	1	1			
Apidae	*Bombus*	*californicus*					1			1		1			
Apidae	*Bombus*	*centralis*					1	1	1	1	1	1	1		
Apidae	*Bombus*	*fervidus*					1	1	1	1	1	1	1		
Apidae	*Bombus*	*flavifrons*						1		1		1			
Apidae	*Bombus*	*huntii*					1	1	1	1	1	1	1		x
Apidae	*Bombus*	*insularis*					1		1	1	1	1	1		
Apidae	*Bombus*	*melanopygus*					1	1		1		1			
Apidae	*Bombus*	*morrisoni*			1	1	1	1	1	1	1	1			
Apidae	*Bombus*	*nevadensis*			1		1	1	1	1	1	1	1		
Apidae	*Bombus*	*occidentalis*					1	1	1	1	1	1	1		x
Apidae	*Bombus*	*rufocinctus*					1		1	1	1	1			
Apidae	*Bombus*	*sylvicola*					1			1		1		*	
Apidae	*Bombus*	*variabilis*							1		1			*	
Apidae	*Centris*	*rhodopus*				1					1				
Apidae	*Ceratina*	*apacheorum*					1			1		1			
Apidae	*Ceratina*	*arizonensis*							1	1		1		*	
Apidae	*Ceratina*	*nanula*				1	1	1	1	1		1	1		
Apidae	*Ceratina*	*neomexicana*					1	1	1	1	1	1			
Apidae	*Ceratina*	*pacifica*				1		1	1	1		1	1		
Apidae	*Ceratina*	001						1		1		1			
Apidae	*Diadasia*	*australis*		1	1	1	1			1		1			
Apidae	*Diadasia*	*diminuta*		1	1	1	1	1	1	1	1	1	1		x
Apidae	*Diadasia*	*enavata*			1		1		1	1		1			
Apidae	*Diadasia*	*ochracea*		1	1	1	1			1	1	1	1		x
Apidae	*Diadasia*	*rinconis*				1	1	1		1		1	1		x
Apidae	*Epeolus*	*compactus*			1					1	1	1			
Apidae	*Epeolus*	*flavofasciatus*					1				1			*	
Apidae	*Epeolus*	*interruptus*					1				1			*	
Apidae	*Epeolus*	*pusillus*					1			1			1	*	
Apidae	*Ericrocis*	*lata*					1				1			*	
Apidae	*Eucera*	*crenulaticornis*					1				1				
Apidae	*Eucera*	*fulvitarsis*	*annae*		1	1				1		1			
Apidae	*Eucera*	*lippiae*					1				1				
Apidae	*Eucera*	*lutziana*		1						1		1			
Apidae	*Eucera*	*ochraea*				1					1			*	
Apidae	*Eucera*	*primiveris*		1	1	1				1		1			
Apidae	*Eucera*	*speciosa*				1				1		1		*	
Apidae	*Eucera*	*territella*			1		1			1		1			
Apidae	*Eucera*	*Eucera* 001					1		1	1		1	1		
Apidae	*Eucera*	*Eucera* 002						1		1		1	1		
Apidae	*Eucera*	*Eucera* 003							1	1			1	*	
Apidae	*Eucera*	(*Synhalonia*) 001					1			1			1		
Apidae	*Exomalopsis*	*solani*		1						1		1			
Apidae	*Exomalopsis*	*solidaginis*					1			1		1		*	
Apidae	*Holcopasites*	*stevensi*				1				1		1		*	
Apidae	*Melecta*	*bohartorum*						1		1		1		*	
Apidae	*Melecta*	*pacifica*		1				1		1		1			
Apidae	*Melissodes*	*agilis*					1				1				
Apidae	*Melissodes*	*bimatris*				1					1			*	
Apidae	*Melissodes*	*coloradensis*									1				
Apidae	*Melissodes*	*communis*				1				1		1		*	
Apidae	*Melissodes*	*compositus*				1					1			*	
Apidae	*Melissodes*	*confusus*					1	1	1	1	1	1	1		
Apidae	*Melissodes*	*coreopsis*					1				1			*	
Apidae	*Melissodes*	*druriellus*					1			1	1	1		*	
Apidae	*Melissodes*	*fasciatellus*		1				1		1			1		
Apidae	*Melissodes*	*gilensis*					1	1	1	1	1	1			
Apidae	*Melissodes*	*glenwoodensis*			1		1			1		1			
Apidae	*Melissodes*	*grindeliae*					1	1	1		1				
Apidae	*Melissodes*	*menuachus*									1				
Apidae	*Melissodes*	*montanus*					1	1		1	1	1	1		
Apidae	*Melissodes*	*pallidisignatus*		1						1	1	1			
Apidae	*Melissodes*	*paroselae*					1				1				
Apidae	*Melissodes*	*perpolitus*				1	1	1		1		1	1		
Apidae	*Melissodes*	*rivalis*					1			1	1	1			
Apidae	*Melissodes*	*saponellus*		1						1		1			
Apidae	*Melissodes*	*semilupinus*		1						1		1		*	
Apidae	*Melissodes*	*tristis*		1	1	1	1	1	1	1	1	1	1		x
Apidae	*Melissodes*	*verbesinarum*				1				1		1			
Apidae	*Nomada*	*texana*									1				
Apidae	*Nomada*	*utahensis*					1		1		1				
Apidae	*Nomada*	*zebrata*					1				1				
Apidae	*Svastra*	*obliqua*		1						1		1		*	
Apidae	*Triepeolus*	*rhododontus*		1			1			1		1			
Apidae	*Triepeolus*	001		1						1		1			
Apidae	*Triepeolus*	003		1						1		1			
Apidae	*Xeromelecta*	*californica*		1			1	1		1	1	1			
Apidae	*Xylocopa*	*californica*					1			1			1		
Colletidae	*Colletes*	*bryanti*			1					1		1		*	
Colletidae	*Colletes*	*compactus*					1			1		1			
Colletidae	*Colletes*	*eulophi*									1				
Colletidae	*Colletes*	*gilensis*					1	1	1	1	1	1	1		
Colletidae	*Colletes*	*kincaidii*					1	1		1	1	1			
Colletidae	*Colletes*	*paniscus*	*paniscus*				1				1				
Colletidae	*Colletes*	*scopiventer*		1	1					1		1			
Colletidae	*Colletes*	*simulans*									1			*	
Colletidae	*Colletes*	*wickhami*		1						1		1			
Colletidae	*Colletes*	*wootoni*					1	1	1		1				
Colletidae	*Colletes*	001						1		1			1		
Colletidae	*Colletes*	002						1		1			1	*	
Colletidae	*Colletes*	003					1			1			1	*	
Colletidae	*Colletes*	004			1					1		1		*	
Colletidae	*Colletes*	005			1					1		1		*	
Colletidae	*Hylaeus*	*annulatus*			1		1	1	1	1	1	1	1		x
Colletidae	*Hylaeus*	*cookii*				1		1	1	1		1	1		
Colletidae	*Hylaeus*	*episcopalis*	*episcopalis*				1	1		1	1	1		*	
Colletidae	*Hylaeus*	*insolitus*					1				1			*	
Colletidae	*Hylaeus*	*rudbeckiae*					1		1	1		1			
Colletidae	*Hylaeus*	*wootoni*					1			1	1	1			
Halictidae	*Agapostemon*	*angelicus*		1	1	1	1	1	1	1	1	1	1		x
Halictidae	*Agapostemon*	*melliventris*		1	1	1				1	1	1			
Halictidae	*Agapostemon*	*texanus*		1	1	1	1	1	1	1	1	1			x
Halictidae	*Dieunomia*	*apacha*									1			*	
Halictidae	*Dieunomia*	*micheneri*				1				1		1			
Halictidae	*Dieunomia*	*nevadensis*		1						1		1		*	
Halictidae	*Halictus*	*confusus*							1	1		1		*	
Halictidae	*Halictus*	*farinosus*		1						1		1		*	
Halictidae	*Halictus*	*ligatus*			1	1	1	1		1	1	1			
Halictidae	*Halictus*	*tripartitus*		1		1	1		1	1	1	1	1		
Halictidae	*Lasioglossum*	*boreale*						1	1	1		1			
Halictidae	*Lasioglossum*	aff.comulum			1		1	1		1		1	1		
Halictidae	*Lasioglossum*	*desertum*			1	1	1	1	1	1	1	1	1		x
Halictidae	*Lasioglossum*	*egregium*		1			1	1	1	1	1	1			
Halictidae	*Lasioglossum*	*hudsoniellum*			1	1		1		1		1			
Halictidae	*Lasioglossum*	*hyalinum*			1					1		1			
Halictidae	*Lasioglossum*	*microlepoides*			1			1		1		1			
Halictidae	*Lasioglossum*	*obnubilum*			1					1		1			
Halictidae	*Lasioglossum*	*occidentale*			1					1		1			
Halictidae	*Lasioglossum*	*pallidellum*			1					1		1			
Halictidae	*Lasioglossum*	cf.perdifficile						1		1		1			
Halictidae	*Lasioglossum*	aff.perparvum			1		1	1		1		1			
Halictidae	*Lasioglossum*	*ruidosense* species-group					1	1	1	1		1			
Halictidae	*Lasioglossum*	*ruficorne*			1		1	1	1	1		1			
Halictidae	*Lasioglossum*	*semicaeruleum*			1	1	1	1		1		1			
Halictidae	*Lasioglossum*	*sisymbrii*		1	1	1	1	1	1	1	1	1	1		x
Halictidae	*Lasioglossum*	new *tegulare* species-group			1					1		1			
Halictidae	*Lasioglossum*	*trizonatum*					1	1		1	1	1	1		
Halictidae	*Lasioglossum*	cf.viridatulum			1			1		1		1			
Halictidae	*Lasioglossum*	001			1		1			1		1			
Halictidae	*Lasioglossum*	002			1					1		1			
Halictidae	*Lasioglossum*	003			1	1				1		1			
Halictidae	*Lasioglossum*	004			1	1	1	1	1	1		1	1		
Halictidae	*Lasioglossum*	005			1		1	1	1	1		1			
Halictidae	*Lasioglossum*	006						1	1	1		1	1		
Halictidae	*Lasioglossum*	007					1	1		1		1			
Halictidae	*Lasioglossum*	008						1		1			1		
Halictidae	*Nomia*	*foxii*				1	1				1				
Halictidae	*Nomia*	*tetrazonata*					1				1			*	
Halictidae	*Protodufourea*	*eickworti*							1	1		1		*	
Halictidae	*Sphecodes*	*pecosensis*		1				1		1		1			
Halictidae	*Sphecodes*	001					1	1		1		1			
Halictidae	*Sphecodes*	002			1					1		1			
Halictidae	*Sphecodes*	003					1			1		1		*	
Halictidae	*Sphecodes*	004						1		1			1	*	
Megachilidae	*Anthidiellum*	*notatum*									1				
Megachilidae	*Anthidium*	*atripes*		1	1					1		1			
Megachilidae	*Anthidium*	*clypeodentatum*					1	1		1	1	1	1		
Megachilidae	*Anthidium*	*cockerelli*		1			1			1		1			
Megachilidae	*Anthidium*	*dammersi*			1					1		1			
Megachilidae	*Anthidium*	*duomarginatum*			1		1			1	1	1			
Megachilidae	*Anthidium*	*emarginatum*					1			1	1	1			
Megachilidae	*Anthidium*	*illustre*				1	1			1	1	1			
Megachilidae	*Anthidium*	*maculifrons*					1				1			*	
Megachilidae	*Anthidium*	*maculosum*				1	1	1		1	1	1			
Megachilidae	*Anthidium*	*mormonum*					1	1		1	1	1			
Megachilidae	*Anthidium*	*palmarum*		1		1				1		1		*	
Megachilidae	*Anthidium*	*porterae*			1	1	1			1	1	1			
Megachilidae	*Anthidium*	*schwarzi*		1						1		1		*	
Megachilidae	*Ashmeadiella*	*aridula*				1				1		1			
Megachilidae	*Ashmeadiella*	*bucconis*				1		1		1		1		*	
Megachilidae	*Ashmeadiella*	*cactorum*	*basalis*	1						1		1			x
Megachilidae	*Ashmeadiella*	*californica*			1		1	1		1	1	1			
Megachilidae	*Ashmeadiella*	*gillettei*		1		1	1	1	1	1		1	1		
Megachilidae	*Ashmeadiella*	*meliloti*		1	1	1	1	1		1		1			
Megachilidae	*Ashmeadiella*	*opuntiae*		1		1	1			1		1			
Megachilidae	*Ashmeadiella*	*sonora*			1	1	1			1		1	1		
Megachilidae	*Ashmeadiella*	*timberlakei*				1	1			1		1			
Megachilidae	*Ashmeadiella*	*vandykiella*		1						1		1		*	
Megachilidae	*Ashmeadiella*	002			1	1				1		1			
Megachilidae	*Atoposmia*	*enceliae*		1						1		1		*	
Megachilidae	*Coelioxys*	*apacheorum*					1				1				
Megachilidae	*Coelioxys*	*erysimi*					1				1			*	
Megachilidae	*Coelioxys*	*gilensis*					1			1	1	1			
Megachilidae	*Coelioxys*	*moestus*					1	1		1	1	1			
Megachilidae	*Coelioxys*	*octodentatus*									1				
Megachilidae	*Coelioxys*	*porterae*					1	1			1				
Megachilidae	*Coelioxys*	*rufitarsis*					1	1		1	1	1			
Megachilidae	*Coelioxys*	*sodalis*					1				1				
Megachilidae	*Coelioxys*	001				1				1		1		*	
Megachilidae	*Coelioxys*	002			1					1		1		*	
Megachilidae	*Coelioxys*	003			1					1		1		*	
Megachilidae	*Dianthidium*	*arizonicum*				1					1			*	
Megachilidae	*Dianthidium*	*concinnum*		1	1	1		1		1		1			
Megachilidae	*Dianthidium*	*cressonii*				1	1	1		1	1	1			
Megachilidae	*Dianthidium*	*curvatum*					1			1		1			
Megachilidae	*Dianthidium*	*desertorum*					1			1		1		*	
Megachilidae	*Dianthidium*	*heterulkei*	*fraternum*			1	1			1	1	1			
Megachilidae	*Dianthidium*	*parvum*	*parvum*	1		1	1			1		1			
Megachilidae	*Dianthidium*	*platyurum*				1				1		1			
Megachilidae	*Dianthidium*	*pudicum*				1	1			1		1			
Megachilidae	*Dianthidium*	*singulare*				1	1			1		1			
Megachilidae	*Dianthidium*	*subparvum*				1	1			1		1			
Megachilidae	*Dianthidium*	*texanum*				1				1		1			
Megachilidae	*Dianthidium*	*ulkei*		1		1	1	1		1		1			
Megachilidae	*Heriades*	*cressoni*				1	1			1	1	1			
Megachilidae	*Heriades*	*gracilior*		1		1				1		1			
Megachilidae	*Heriades*	*micropthalma*									1				
Megachilidae	*Heriades*	*texana*									1			*	
Megachilidae	*Heriades*	*timberlakei*			1	1				1		1			
Megachilidae	*Heriades*	002						1		1		1			
Megachilidae	*Hoplitis*	*grinnelli*									1				
Megachilidae	*Hoplitis*	*paroselae*		1						1		1		*	
Megachilidae	*Hoplitis*	*truncata*	*mescalerium*				1				1				
Megachilidae	*Hoplitis*	*zuni*				1				1		1			
Megachilidae	*Hoplitis*	001		1						1		1		*	
Megachilidae	*Lithurgopsis*	*apicalis*		1		1	1			1	1	1			
Megachilidae	*Lithurgopsis*	*planifrons*				1	1			1					
Megachilidae	*Megachile*	*agustini*									1			*	
Megachilidae	*Megachile*	*angelarum*							1	1	1	1	1		
Megachilidae	*Megachile*	*brevis*									1				
Megachilidae	*Megachile*	*chilopsidis*				1				1		1		*	
Megachilidae	*Megachile*	*comata*			1		1	1	1	1	1	1	1		
Megachilidae	*Megachile*	*fidelis*					1	1	1	1	1	1			
Megachilidae	*Megachile*	*fortis*							1	1	1	1			
Megachilidae	*Megachile*	*frigida*					1	1	1	1	1	1	1		
Megachilidae	*Megachile*	*inimica*	*sayi*				1	1		1	1	1			
Megachilidae	*Megachile*	*lapponica*						1		1		1			
Megachilidae	*Megachile*	*latimanus*							1	1		1			
Megachilidae	*Megachile*	*lippiae*		1						1	1	1			
Megachilidae	*Megachile*	*lobatifrons*		1						1		1		*	
Megachilidae	*Megachile*	*manifesta*									1			*	
Megachilidae	*Megachile*	*melanophaea*						1		1	1	1			
Megachilidae	*Megachile*	*mellitarsis*				1	1	1	1	1	1	1	1		
Megachilidae	*Megachile*	*montivaga*		1		1	1	1		1	1	1			
Megachilidae	*Megachile*	*mucida*					1				1			*	
Megachilidae	*Megachile*	*mucorosa*			1					1		1			
Megachilidae	*Megachile*	*onobrychidis*			1	1				1		1			
Megachilidae	*Megachile*	*perihirta*					1				1				
Megachilidae	*Megachile*	*policaris*		1						1	1	1			
Megachilidae	*Megachile*	*pugnata*	*pomonae*				1	1		1	1	1			
Megachilidae	*Megachile*	*relativa*		1	1		1	1	1	1	1	1	1		
Megachilidae	*Megachile*	*sabinensis*		1						1		1			
Megachilidae	*Megachile*	*sidalceae*		1						1		1		*	
Megachilidae	*Megachile*	*snowi*					1	1			1				
Megachilidae	*Megachile*	*subexilis*					1	1		1	1	1			
Megachilidae	*Megachile*	*sublaurita*		1	1	1	1			1	1	1			
Megachilidae	*Megachile*	*texana*									1				
Megachilidae	*Megachile*	001					1			1		1		*	
Megachilidae	*Megachile*	002						1		1		1		*	
Megachilidae	*Osmia*	*albolateralis*					1				1				
Megachilidae	*Osmia*	*brevis*					1	1		1			1		
Megachilidae	*Osmia*	*bucephala*					1	1	1	1		1			
Megachilidae	*Osmia*	*coloradensis*		1			1	1		1	1	1	1		
Megachilidae	*Osmia*	*densa*					1				1				
Megachilidae	*Osmia*	*juxta*					1	1	1	1		1	1		
Megachilidae	*Osmia*	*lignaria*					1				1				
Megachilidae	*Osmia*	*liogastra*		1						1		1		*	
Megachilidae	*Osmia*	*montana*							1	1		1		*	
Megachilidae	*Osmia*	*pentstemonis*					1				1				
Megachilidae	*Osmia*	aff.pentstemonis							1	1		1	1		
Megachilidae	*Osmia*	*simillima*					1	1	1	1	1	1	1		
Megachilidae	*Osmia*	*subaustralis*							1	1	1	1			
Megachilidae	*Osmia*	*texana*			1		1	1	1	1	1	1			
Megachilidae	*Osmia*	*unca*					1				1			*	
Megachilidae	*Osmia*	002						1	1	1		1			
Megachilidae	*Osmia*	003						1		1		1		*	
Megachilidae	*Osmia*	005					1		1	1		1			
Megachilidae	*Osmia*	006							1	1		1		*	
Megachilidae	*Osmia*	007							1	1		1		*	
Megachilidae	*Osmia*	009						1	1	1		1			
Megachilidae	*Osmia*	010						1	1	1		1			
Megachilidae	*Osmia*	011						1		1		1		*	
Megachilidae	*Osmia*	012					1		1	1			1		
Megachilidae	*Osmia*	(*Cephalosmia*) 001						1		1		1			
Megachilidae	*Osmia*	(*Cephalosmia*) 002							1	1		1		*	
Megachilidae	*Osmia*	(*Melanosmia*) 001						1		1			1	*	
Megachilidae	*Osmia*	(*Melanosmia*) 002					1			1		1		*	
Megachilidae	*Paranthidium*	*jugatorium*		1		1	1	1		1	1	1	1		
Megachilidae	*Stelis*	*elegans*					1			1			1	*	
Megachilidae	*Stelis*	*rudbeckiarum*					1			1	1	1			
